# Distinct Microbial Communities in Adjacent Rock and Soil Substrates on a High Arctic Polar Desert

**DOI:** 10.3389/fmicb.2020.607396

**Published:** 2021-01-08

**Authors:** Yong-Hoe Choe, Mincheol Kim, Yoo Kyung Lee

**Affiliations:** Korea Polar Research Institute, Incheon, South Korea

**Keywords:** polar desert, lithic niche, edaphic niche, rock microbes, Arctic

## Abstract

Understanding microbial niche variability in polar regions can provide insights into the adaptive diversification of microbial lineages in extreme environments. Compositions of microbial communities in Arctic soils are well documented but a comprehensive multidomain diversity assessment of rocks remains insufficiently studied. In this study, we obtained two types of rocks (sandstone and limestone) and soils around the rocks in a high Arctic polar desert (Svalbard), and examined the compositions of archaeal, bacterial, fungal, and protistan communities in the rocks and soils. The microbial community structure differed significantly between rocks and soils across all microbial groups at higher taxonomic levels, indicating that Acidobacteria, Gemmatimonadetes, Latescibacteria, Rokubacteria, Leotiomycetes, Pezizomycetes, Mortierellomycetes, Sarcomonadea, and Spirotrichea were more abundant in soils, whereas Cyanobacteria, Deinococcus-Thermus, FBP, Lecanoromycetes, Eurotiomycetes, Trebouxiophyceae, and Ulvophyceae were more abundant in rocks. Interestingly, fungal communities differed markedly between two different rock types, which is likely to be ascribed to the predominance of distinct lichen-forming fungal taxa (Verrucariales in limestone, and Lecanorales in sandstone). This suggests that the physical or chemical properties of rocks could be a major determinant in the successful establishment of lichens in lithic environments. Furthermore, the biotic interactions among microorganisms based on co-occurrence network analysis revealed that *Polyblastia* and *Verrucaria* in limestone, and *Atla*, *Porpidia*, and *Candelariella* in sandstone play an important role as keystone taxa in the lithic communities. Our study shows that even in niches with the same climate regime and proximity to each other, heterogeneity of edaphic and lithic niches can affect microbial community assembly, which could be helpful in comprehensively understanding the effects of niche on microbial assembly in Arctic terrestrial ecosystems.

## Introduction

Understanding the microbial community structure and diversity in polar regions is fundamentally important in both microbial ecology and evolution (Allen and Banfield, 2005). The microbial colonization found in these habitats not only provides insights into microbial succession on uncolonized niches such as glacial forelands, but also helps understand the adaptive mechanisms to the extreme environment for their stressful factors. Studies to identify the diversity and dynamics of the microbial community have been conducted in a variety of extreme terrestrial ecosystems, including the tundra region (McCann et al., 2016), high-altitude areas (Wong et al., 2010; Tolotti et al., 2020), hyper-arid deserts (Wierzchos et al., 2006, 2013), and Antarctic deserts (de los Rios et al., 2005; Lee et al., 2019). In the case of high Arctic regions, although the distribution pattern and function of edaphic microbial communities have been extensively documented (Kastovska et al., 2005; Schutte et al., 2009; Wilhelm et al., 2011; Tveit et al., 2013; Blaud et al., 2015; McCann et al., 2016), few studies have investigated lithic microbial communities, and these have mainly focused on bacteria and/or fungi (Omelon et al., 2006, 2007; Omelon, 2008; Ziolkowski et al., 2013; Choe et al., 2018). These studies suggest that rocks can be a hotspot for microbial diversity and that the physical and chemical properties of lithic substrates affect microbial colonization. However, there have been few comprehensive multidomain biodiversity assessments on polar regions (Pointing et al., 2009; Garrido-Benavent et al., 2020), and variations in the microbial diversity between different niches (rocks and soils) are still poorly understood.

The climate regime plays a major role in determining the diversity and dynamics of microbial communities, and niche characteristics can also be one of the crucial factors affecting phylogenetic diversity as well as their survival strategies, especially in polar regions (Pointing and Belnap, 2012). Several studies have attempted to demonstrate the microstructure, physicochemical properties, and nutrient availability of the habitat, which are thought to be determinants of microbial diversity and survival strategies (Rogers and Bennett, 2004; Carson et al., 2009). In the case of lithic habitat, the microhabitat architecture of the rock, such as translucence, thermal conductivity, the pore network, and chemical composition is one of main drivers of lithic microbial colonization (Wierzchos et al., 2015). Furthermore, rock weathering in the early stages of soil development in the polar regions, such as the glacial foreland, can promote certain microbial communities in the surrounding soil by leaching of rock minerals (Borin et al., 2010). Similarly, soils are also heterogeneous habitats, containing a variety of spatial scales and environmental gradients (Lehmann et al., 2008). These characteristics provide a large potential for niche differentiation and may be an important factor in developing the diversity of microbial communities in soil (Young et al., 2008). These studies have shown distinct microbial communities that are specialized in soil and rock, respectively. However, it remains unexplored whether the differences in microbial composition are also observed in an adjacent edaphic and lithic niche.

In this s tudy, we examined the diversity and structure of four microbial groups (archaea, bacteria, fungi, and protists) of two selected rocks (sandstone and limestone) and two types of soils around the limestone and sandstone each, and then confirmed whether these groups were specialized in niches. We conducted the test in the same climate regime to minimize the impact of climate variability. Soil and rock chemical characteristics were analyzed to account for the differences in niches. Furthermore, microstructural analyses of rock were used to identify the differences in physical properties between the two types of rock and the potential contributions of structural differences to lithic microbial colonization. This study offers a holistic view of edaphic and lithic microbial communities and helps understand the relationship between niche characteristics and microbial community structure in a polar desert.

## Materials and Methods

### Field Sampling

**FIGURE 1:**
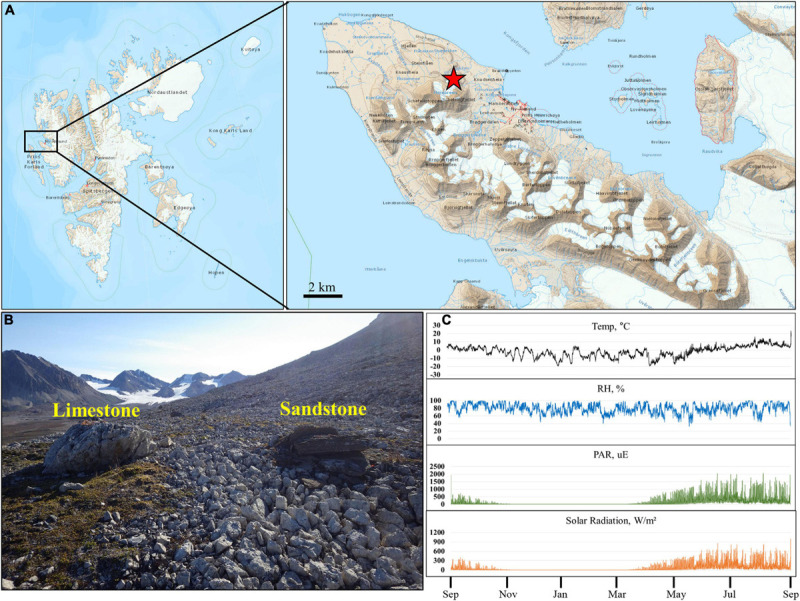
Location and microclimate data of the study site. **(A,B)** Present location and landscape of the sampling sites. **(C)** Present annual temperature, relative humidity, photosynthetically active radiation, and total solar radiation of the study site. The map was prepared based on the map of TopoSvalbard made by the Norwegian Polar Institute.

The sampling site was located in an exposed knoll about 4 km NW of Ny-Ålesund in Svalbard. The site was composed mainly of dolomitic limestone and sandstone of the Carboniferous age (Figures 1A,B). A total of 76 samples consisting of limestone (Lime), sandstone (Sand), soil around limestone (Lime-Soil), and soil around sandstone (Sand-Soil) were collected from the site in August 2016 (Supplementary Table 1). For rock samples, three different parts of each rock were randomly sampled and pooled into a sterile plastic bag without discriminating the surface and inner parts. In the case of soil samples, soils at three points around each rock sample were pooled into a sterile plastic bag. The samples were placed in a cooler with ice packs during transport to the laboratory at Dasan Research Station, and then stored at −20°C until analysis. Soil samples were not sieved, but gravels larger than 3–4 cm size were removed. Rock samples were crushed using a Mixer Mill (Retsch, Germany) for genomic DNA extraction.

### Microclimate Data

The climate data of the study area were recorded during every 10 min intervals using an Onset HOBO^®^ Micro weather Station Data Logger (H21-002), from August 10th, 2015 to July 25th, 2016. Relative humidity (RH) and air temperature (T) were recorded using RH/T sensors (HOBO^®^ S-THB-M002) shaded from the sun 100 cm above the rock surface. Solar flux was measured using a quantum sensor (S-LIA-M003 and S-LIB-M003) for photosynthetic active radiation (PAR) and total solar radiation.

### Major and Trace Element Analysis

The major element compositions of the rock and soil samples were determined using a wavelength-dispersive XRF spectrometer (Panalytical, Netherlands) at the Korea Polar Research Institute. Three glass disks per sample prepared by fusing a powdered sample with LT 100 flux (100% Li tetraborate; XRF Scientific, Australia) were used for XRF analysis. Each disk was measured three times, and the average value was taken. To determine trace element concentrations of rocks and soils, finely ground samples were dissolved by acid digestion. The concentration of the final solutions was determined using an inductively coupled plasma mass spectrometry (ICP-MS) using a Thermo ICAP-Q mass spectrometer at the Korea Polar Research Institute.

### Pore Structure of Lithic Substrates

The pore size distribution and porosity of limestone and sandstone were characterized by mercury intrusion porosimetry (MIP) at the Korea Basic Science Institute using an AutoPore IV 9500 (Micromeritics Instrument, Norcross, United States) instrument. Three subsamples were prepared for each rock sample, and each subsample was measured three times.

X-ray CT was used to characterize three-dimensional images of the pore structure and distribution in the subsurface of the rock samples. Three cylindrical samples (average height of 20 mm) were cut from limestone (*n* = 3) and sandstone (*n* = 3). X-ray CT analysis was carried out using an X-EYE system at the Korea Institute of Civil Engineering and Building Technology. Pore images were acquired using an adaptive thresholding technique based on X-ray CT images.

### Total Water Retention Capacity

Rock samples of approximately 5 cm^3^ were weighed prior to immersion in H_2_O, and then the rock samples were immersed in H_2_O. After 24 h, the rock samples were weighed again by removing the excess gravitational water. The total water retention capacity (TWRC) is shown as (%) w/w of retained water per g of rock. Triplicates were performed for limestone and sandstone.

### Processing of Illumina Amplicon Sequence Data

Total DNA was extracted from 3 g of powdered rock and 3 g of soil using the FastDNA SPIN kit (MP Biomedicals, Illkirch, France), following the manufacturer’s protocol. DNA samples were submitted for PCR amplification, library preparation, and paired-end Illumina MiSeq sequencing (2 × 300 bp) to the Integrated Microbiome Resource (IMR) facility at the Centre for Genomics and Evolutionary Bioinformatics at Dalhousie University (Halifax, Canada). The extracted DNA was amplified using primers 956F and 1401R targeting the V6-V8 region of the archaeal 16S rRNA gene (Comeau et al., 2011), the primer pair 515F-926R targeting the V4-V5 region of the bacterial 16S rRNA gene (Parada et al., 2016; Walters et al., 2016), the primer pair 572F-1009R targeting the V4 region of the eukaryotic 18S rRNA gene (Comeau et al., 2011), and the pair ITS86F-TS4R targeting the ITS2 region of the fungal nuclear ribosomal internal transcribed spacer (De Beeck et al., 2014). Samples that were not amplified were excluded from further analysis (see Supplementary Table 1).

Quality control analysis of the paired-end reads was conducted to determine the length of the reads that can be trimmed in downstream steps, using FastQC^1^. Cutadapt v2.10 was run to remove the remaining adapter and primer sequences from both ends (Martin, 2011). The sequence reads were processed following the QIIME2 pipeline (version 2018.2 platform)^2^ (Bolyen et al., 2018). Briefly, the sequence reads of archaeal 16S rRNA genes (at base position 240 and 200 bp for forward and reverse reads, respectively), bacterial 16S rRNA genes (270 and 230 bp), fungal ITS2 regions (250 and 220 bp), and eukaryotic 18S rRNA genes (260 and 220 bp) were trimmed to discard base pairs with average Phred quality score less than 30 and dereplicated using DADA2 (Callahan et al., 2016) as implemented in QIIME2 with paired-end setting (including quality control, trimming, pair-joining, and chimera removals). The QIIME2 q2-feature-classifier^3^, a naive Bayes classifier, was used to assign taxonomy. All ASVs were assigned a taxonomic classification using the Silva database (release 128, Quast et al., 2013) for archaeal and bacterial 16S rRNA gene, the UNITE database (v8.0, Abarenkov et al., 2010) as a reference database for fungal ITS2 region, and the PR^2^ database (v.4.10.0, Guillou et al., 2013) as a reference database for the 18S rRNA gene. To selectively analyze protistan communities in the 18S rRNA sequence data, the sequences assigned to the fungal 18S rRNA gene were filtered out prior to further analyses.

### Statistical Analyses

Alpha diversity, including richness, Shannon and Simpson indices, and evenness of rock and soil samples were calculated using QIIME2 (version 2018.2 platform; see text footnote 2) with subsampling depth based on the lowest sequences of sample for each domain. A one-way between subjects ANOVA and *post hoc* comparison using the Duncan’s LSR test were conducted to compare relative abundance among the niches for all domains at the phylum (or class) level. Where assumptions for parametric tests were not met, a Kruskal-Wallis test for independent samples and a Dunn’s *post hoc* test were performed (R’s agricolae and FSA package). Non-metric multidimensional scaling (NMDS) ordinations were constructed on the basis of Bray-Curtis dissimilarities to demonstrate differences in the composition of microbial communities across sample groups. The differences in microbial community structure between two substrates (rock and soil) were identified by analysis of similarities (ANOSIM) (Clarke, 1993). The effects of substrates and rock types (limestone and sandstone) and their interaction on the microbial dissimilarity were tested by a two-way permutational multivariate analysis of variance using the Adonis function in the Vegan package^4^. We also tested for differences in the dispersion of substrates by performing an analysis of multivariate homogeneity [PERMDISP, (Anderson, 2006)] with the function “betadisper” implemented in the R package “vegan” (Oksanen et al., 2016) using default parameters. ASVs that predominantly contributed to these differences were identified using similarity percentage (SIMPER) analysis using PRIMER-E (Clarke and Gorley, 2006). Furthermore, we identified the “niche breadth” using the formula

$$B_{j}=\frac{1}{\sum_{i}^{N}P_{i\,j}^{2}}$$

where *B*_*j*_ is the niche breadth and *P*_*ij*_ indicates the proportion of individuals of species *j* found in a given niche *i*. ASVs that were more evenly distributed along a wider range of niches were considered as generalists, whereas ASVs with a lower *B*-value were regarded as specialists. Niche breadth was calculated for the 500 ASVs with the highest mean relative abundance. The values for niche breadth ranged from 1 to 25. ASVs with B >3.5 were regarded as generalists, and ASVs with B < 1.5 were considered specialists, which were identified according to the approach by Logares et al. (2013) and Maier et al. (2018).

Co-occurrence networks for lithic niches were constructed using the SParse InversE Covariance Estimation for Ecological Association Inference (SPIEC-EASI) for combined bacterial and fungal datasets (Kurtz et al., 2015; Tipton et al., 2018). To reduce the complexity of network visualization, rare ASVs were filtered out by defining thresholds of a relative abundance >0.001% and a minimum of six occurrences in a dataset consisting of samples that recovered both bacteria and fungi (18 limestone samples and 18 sandstone samples). We used the MB (Meinshausen and Bühlmann) method as an inference scheme, and the StARS variability threshold was set to 0.05 for all networks. The topological properties of networks were analyzed using the package “igraph” (Csardi and Nepusz, 2006). We considered ASVs with highest betweenness centrality in the network as keystone taxa (Martin González et al., 2010; Vick-Majors et al., 2014; Banerjee et al., 2016).

## Results

### Microclimate Data

Microclimatic parameters such as relative humidity, photosynthetically active radiation (PAR), and air temperature were recorded for 12 months, from August 2015, at close proximity to the sampling site (Figure 1C). Solar radiation and PAR values showed a similar pattern throughout the year. During the summer period, the spikes of solar radiation and PAR values were up to 1004 W/m^2^ and 2066 μE, respectively. The fluctuation in annual air temperatures was in accordance with seasonal changes in solar irradiance. The annual mean temperature for the 12 month period was −1.8°C. The mean daily maximum temperature in summer (June-August) was 10.2°C with a maximum of 23.3°C. The minimum recorded air temperature was −19.7°C in January. Relative humidity rarely fell below 40%, rose above 60% during a year, and occasionally reached 100%.

### Chemical and Physical Characterization of Niches

The major and trace element compositions of selected rock (Limestone and Sandstone) and soil (Lime-Soil and Sand-Soil) samples are shown in Supplementary Table 3. In limestone, Ca and Mg were relatively high, and the content of Sr was the highest among the trace elements. In contrast, the content of Si was significantly higher in sandstone, and V, Cr, Zn, Rb, Sr, and Ba were also abundant in sandstone. In soil, Lime-Soil and Sand-Soil showed similar elemental distributions with relatively high contents of Si, Ca, V, Cr, Zn, Rb, Sr, and Ba, which were significantly different from limestone but similar to that of sandstone.

X-ray computerized tomography (CT) was used to better characterize the subsurface microstructure of limestone and sandstone (Supplementary Figure 1). The resulting X-ray CT scans showed a different pore distribution in the subsurface between limestone and sandstone. In terms of the spatial distribution of pores, the pores of the sandstone were distributed mainly from the surface to a depth of 5 mm (Supplementary Figure 1A). Conversely, in the limestone, the pores were evenly distributed throughout (Supplementary Figure 1D). These distributions correspond to longitudinal section images with pores marked in black spots (Supplementary Figures 1B,E). These results were also observed in the graph of porosity change with height (Supplementary Figures 1C,F).

Mercury intrusion porosimetry (MIP) analysis was conducted to analyze the distribution of smaller pores (Supplementary Table 4). The results showed that the limestone has a greater mean pore diameter (294.333 ± 49.460 nm) and median pore diameter (2100.867 ± 123.727 nm) than sandstone. However, the porosities of limestone and sandstone were not significantly different. In addition, the total water retention capacity (TWRC) was measured as a feature related to the difference in the physical structure of the rock. Although the difference in TWRC between limestone and sandstone was statistically significant, it was very small.

### Microbial Community Composition

Rock and soil samples showed distinct differences in relative abundance and microbial diversity under the same climatic regime. After the quality filtering processes on positive amplifications were completed, we obtained a total of 456432, 924726, 980868, and 239390 high-quality sequences for archaea, bacteria, fungi, and protists, respectively (Supplementary Tables 1, 2). Rarefaction curves approached plateau at their respective maximum sampling depths, indicating that sequencing depth was sufficient to identify the majority of ASVs within microbial communities at each niche (Supplementary Figure 2).

**FIGURE 2:**
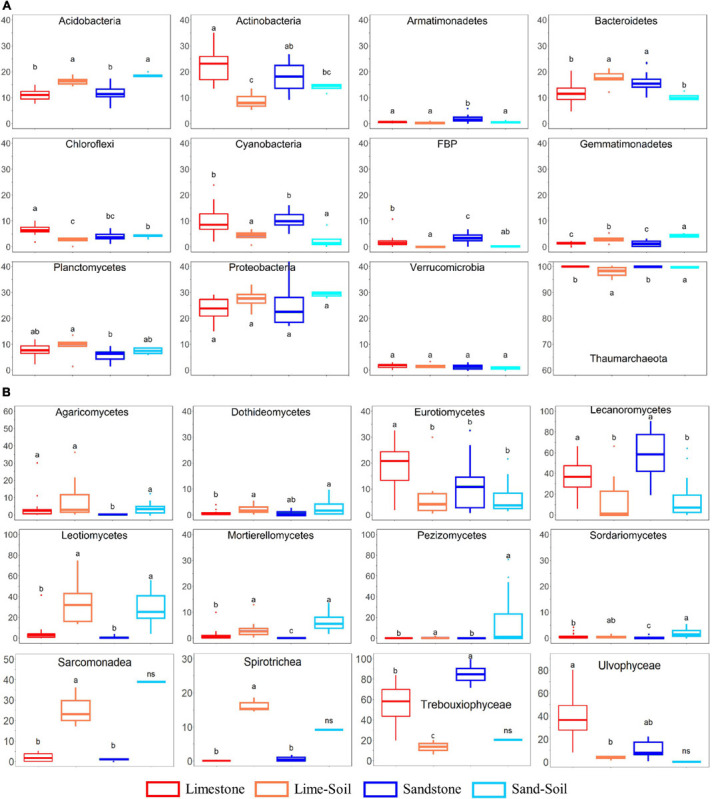
The relative abundance of bacterial, archaeal, fungal, and protistan taxa in four different niches. **(A)** Bacterial and archaeal taxa; **(B)** fungal and protistan taxa. Boxes limit the 25th- and 75th percentile with the median presented as the line. Error bars present the 1st and 99th percentile and outliers are shown as dots below and above. Significant differences (*P* < 0.05) are marked by different letters (see also Supplementary Table 5).

The most abundant archaeal phyla were Thaumarchaeota (average: 99 ± 1.35% across all samples) with small contributions from Euryarchaeota (average: 1 ± 1.35% soil samples only) (Figure 2A and Supplementary Table 5). At the class level, Nitrososphaera (100 ± 1.03%) mainly contributed across all niches and Nitrososphaera were primarily represented by the order Nitrososphaerales (100 ± 1.03%). Although there was a significant difference in community composition between edaphic and lithic niches, we could not find any taxonomic differences at a fine level due to the limitation of taxonomic assignment at a lower level.

A total of 28 bacterial phyla were identified in the edaphic and lithic communities. Proteobacteria was the most abundant phylum across the entire sample set (average: 24.7 ± 5.4% across all samples), followed by Actinobacteria (18.3 ± 6.6%), Bacteroidetes (13.8 ± 4.1%), Acidobacteria (12.5 ± 3.4%), Cyanobacteria (8.8 ± 4.8%), Planctomycetes (7.0 ± 2.6%), Chloroflexi (4.8 ± 2.1%), and FBP (2.2 ± 2.1%) (Figure 2A and Supplementary Table 5). At the class level, Alphaproteobacteria (16.4 ± 6.3%), Bacteroidia (12.5 ± 4.2%), Thermoleophilia (11.9 ± 5.4%), Oxyphotobacteria (8.8 ± 4.8%), Gammaproteobacteria (7.9 ± 5.3%), Blastocatellia (5.6 ± 2.7%), Planctomycetacia (4.1 ± 1.9%), and Actinobacteria (3.6 ± 1.9%) were highly abundant. We also observed statistically significant differences in the relative abundance between the lithic and soil niches (mean relative abundance >1%; Figure 2A and Supplementary Table 5). The relative abundances of Acidobacteria, Gemmatimonadetes, Latescibacteria, and Rokubacteria were significantly higher in soil samples than in rock samples, whereas the relative abundance of Cyanobacteria, Deinococcus-Thermus, and FBP were significantly higher in rock samples than in soil samples.

Across all samples, the most abundant fungal classes were Lecanoromycetes (average: 34.5 ± 25.9%), unclassified fungi (26.7 ± 15.6%), Leotiomycetes (13.9 ± 18.1%), and Eurotiomycetes (12.0 ± 10.8%), with smaller contributions from Pezizomycetes (3.5 ± 13.9%), Agaricomycetes (3.4 ± 6.7%), Mortierellomycetes (2.3 ± 3.3%), and Dothideomycetes (1.9 ± 1.8%). At the order level, Lecanorales (26.5 ± 23.2%), unclassified Ascomycota (18.2 ± 12.2%), Verrucariales (11.5 ± 10.9%), unclassified fungi (8.2 ± 7.3%), Thelebolales (6.9 ± 11.7%), Helotiales (6.7 ± 11.2%), and Pezizales (3.5 ± 13.9%) mainly contributed to the fungal community. The relative abundances of Leotiomecetes, Pezizomycetes, and Mortierellomycetes were significantly higher in edaphic niches than in lithic niches, whereas the relative abundance of Lecanoromycetes and Eurotiomycetes were significantly higher in lithic niches than in edaphic niches (Figure 2B and Supplementary Table 5). In lithic fungal communities, Eurotiomycetes and Lecanoromycetes were most abundant in both limestone and sandstone, but the relative abundance at the order level was different. Eurotiales, Hymeneliales, and Verrucariales were more abundant in limestone communities than in sandstone communities, but the relative abundance of Acarosporales, Candelariales, Lecanorales, Lecideales, Ostropales, Peltigerales, Rhizocarpales, and Umbilicariales were higher in the latter (Supplementary Figure 3).

The inferred protistan ASVs were mainly assigned to the phyla Chlorophyta (average: 76.0 ± 31.5% across all samples), followed by Cercozoa (11.5 ± 16.2%), Ciliophora (5.2 ± 7.4%), and Ochrophyta (1.9 ± 4.2%). At the class level, we also observed statistically significant differences in the relative abundance between the lithic and edaphic niches (mean relative abundance >5%; Figure 2B and Supplementary Table 5). The relative abundances of Sarcomonadea and Spirotrichea were significantly higher in lithic niches than in edaphic niches, whereas the relative abundances of Trebouxiophyceae and Ulvophyceae were significantly higher in edaphic niches.

Alpha diversity indices, such as richness, and Shannon and Simpson indices tended to be higher in edaphic than in lithic archaeal, bacterial, and protistan communities (Supplementary Figure 4). For fungi, these indices were comparable for both niches, except for ASV richness which was higher in lithic than in edaphic communities. The evenness of edaphic communities of bacteria and protists was higher than that of lithic communities, whereas archaeal and fungal communities were similar in both niches (Supplementary Figure 4).

### Niche Differentiation of Microbial Communities in Rock and Soil

**FIGURE 3:**
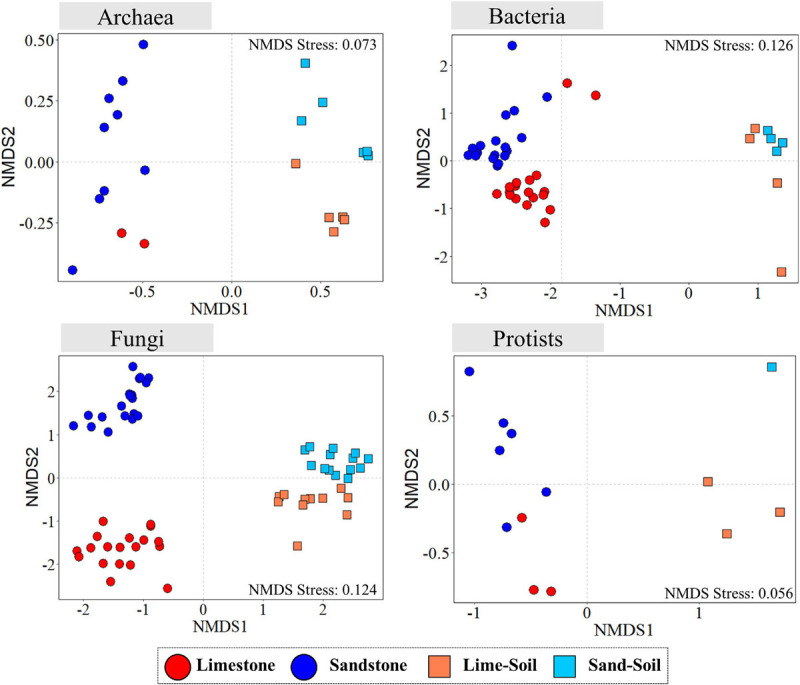
Non-metric multidimensional scaling (NMDS) ordination plots of Bray–Curtis dissimilarities for archaeal, bacterial, fungal, and protistan communities across sample categories.

The community structure of all four microbial groups (bacteria, archaea, fungi, and protists) differed mainly by substrate type (rock vs. soil), followed by rock type (limestone vs. sandstone) (Figure 3 and Supplementary Table 6). The similarity percentage analysis (SIMPER) was used to determine the relative contribution of an individual ASV to the community dissimilarity among the niches (Supplementary Table 7). To achieve the higher taxonomic resolution of those ASVs, the ASVs were further BLAST-searched against the NCBI nucleotide collection database (nr/nt). For archaea, the average dissimilarity between lithic and edaphic niches was 71.98% and was driven by ASV062 (contribution to dissimilarity: 10.4%), ASV050 (7.0%), and ASV031 (6.8%). The average dissimilarity between limestone and sandstone was 52.16% and was driven by ASV036 (11.4%), ASV075 (8.4%), and ASV062 (7.3%). For bacteria, the members of the Gemmatimonadetes contributed to 1.24–1.56% of the dissimilarity of bacterial communities between the lithic niches and soil niches. Although many bacterial phyla consistently contributed to community dissimilarity between limestone and sandstone, the top 3 ASVs that contributed the most to community dissimilarity were the members of Actinobacteria and Chloroflexi (0.40–0.46%). Among the ASVs largely responsible for the dissimilarity of fungal communities between rock and soil, the top 3 genera were *Geomyces* (3.36%), *Pulvinula* (1.07%), and *Mortierella* (1.03%) (Supplementary Table 7). In lithic niches, the ASVs belonging to Lecanoromycetes contributed the most to the dissimilarity between limestone and sandstone. In protistan communities, although there was the limitation of taxonomic assignment at a lower level, the ASVs, which contributed to the dissimilarity of protistan communities, were members of Cercozoa and Chlorophyta (Supplementary Table 7). Furthermore, the results of the PERMDISP analysis revealed differentiation among edaphic and lithic bacterial and fungal communities, indicating that lithic bacterial and fungal communities were significantly more variable in their intra-ASV composition than the edaphic ones (Supplementary Figures 5, 6).

**FIGURE 4:**
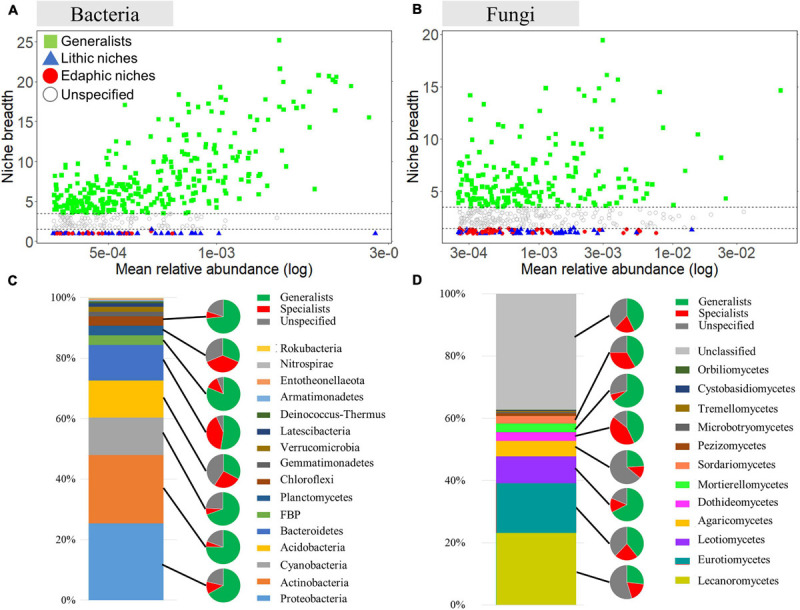
Analysis of niche specialists and generalists. Niche breadth of bacterial ASVs **(A)** and fungal ASVs **(B)** identified in lithic and edaphic niches. Each symbol represents an ASV. ASVs with a *B* value >3.5 are considered niche generalists, while ASVs with a *B* value < 1.5 are considered niche specialists. Phylum compositions of all taxa, specialists, generalists are shown. The bar graph shows the phylum compositions of total bacteria **(C)** and fungi **(D)**. The smaller pies show the proportions of specialists, generalists and unspecified ASVs.

For bacteria and fungi, in which the number of recovered samples was sufficient and well assigned to the lower taxonomic level, we performed further analysis to identify generalists and specialists between rocks and soils. The analyses used the 500 ASVs with the highest mean relative abundance in rock and soil samples. For bacteria, we identified 304 ASVs (out of 500 ASVs, 60.8%) as generalists and 84 ASVs as specialists (16.8%) (Figures 4A,C). Among bacterial generalists, ASVs belonging to Proteobacteria (85 ASVs) and Actinobacteria (85 ASVs) were the most abundant phyla, followed by Cyanobacteria (43 ASVs), Bacteroidetes (31 ASVs), and Acidobactera (20 ASVs). Among all specialists, ASVs belonging to Bacteroidetes (24 ASVs), Acidobacteria (16 ASVs), and Proteobacteria (15 ASVs) were dominant. A much higher proportion of Proteobacteria, Actinobacteria, Cyanobacteria, FBP, and Chloroflexi was present within generalists than specialists, whereas the proportion of specialists in Acidobacteria, Bacteroidetes, and Planctomycetes was higher or similar to that of generalists. The specialists belonging to Bacteroidetes were most abundant in lithic niches, whereas Proteobacteria were more abundant in the edaphic niches (Supplementary Table 9). For fungi, among 500 ASVs, 198 generalist (39.6%) and 98 specialist ASVs (19.6%) were detected (Figures 4B,D). In all taxa, Lecanoromycetes were detected as the most abundant classes (except unclassified 185 ASVs), followed by Eurotiomycetes (79 ASVs), Leotiomycetes (43 ASVs), Agaricomycetes (25 ASVs), Dothideomycetes (14 ASVs), Mortierellomycetes (14 ASVs), Sordariomycetes (12 ASVs), and Pezizomycetes (4 ASVs). Among generalist ASVs, members of Eurotiomycetes, Lecanoromycetes, and Leotiomycetes were the most abundant. The proportion of generalists was higher than that of specialists in most taxa, except for Dothideomycetes. The ASVs belonging to Eurotiomycetes and Lecanoromycetes were the most abundant as a specialist in lithic niches, whereas most abundant specialists in edaphic niches were Leotiomycetes (Supplementary Table 8).

### Co-occurrence Patterns of Bacterial and Fungal Communities in Lithic Niches

**FIGURE 5:**
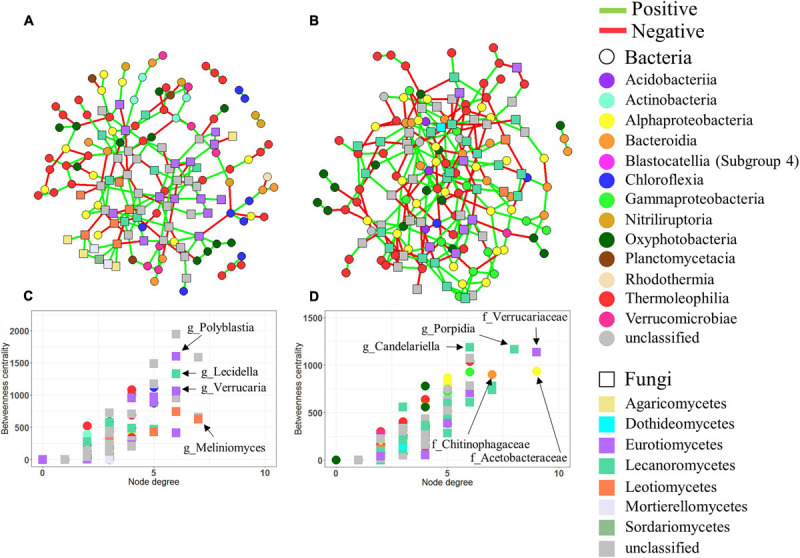
Co-occurrence networks. Networks based on bacterial and fungal ASVs combined in limestone **(A)** and sandstone **(B)**. In all networks, circles and squares correspond to bacterial and fungal ASVs, respectively, with colors representing each taxonomic rank, class. Edges represent positive (green) or negative (red) interactions. Betweenness centrality and degree of each ASV in the limestone network **(C)** and sandstone network **(D)**. Nodes with high betweenness centrality and high degree values are considered to be keystone taxa in the networks. The genera of the bacterial and fungal ASVs with the highest degree and centrality are shown.

Limestone and sandstone showed clear differences in terms of their physical and chemical properties (Supplementary Figure 1 and Supplementary Table 3). Moreover, the microbial community composition between limestone and sandstone also differed significantly. We further conducted a network analysis to identify the difference in the biotic interactions among microorganisms between two types of rocks. Two networks were generated by rock type (Figure 5), and both limestone and sandstone networks were very sparse, with an average node degree of 2.699 and 3.248, respectively. The modularity, clustering coefficient, average path length, positive/negative edge ratio, and network diameter of the limestone network were greater than those of the sandstone network (Supplementary Table 9). In terms of interactions between taxa in the network, the number of intra-kingdom links in bacterial taxa or fungal taxa was higher than the number of inter-kingdom links between bacterial and fungal taxa (Supplementary Table 10).

Among ASVs identified as keystone taxa (i.e., the largest number of degrees and greatest betweenness) for each network, most of them were affiliated with lichen-forming fungal taxa in both limestone and sandstone, but the taxonomic affiliations at the genus level of those ASVs differed depending on the rock type. The keystone fungal ASVs within the limestone included genera *Polyblastia* and *Verrucaria*, whereas the keystone ASVs within sandstone were assigned to genera such as *Atla*, *Porpidia*, and *Candelariella* (Supplementary Table 11). The bacterial taxa that appeared to be positively correlated with fungal keystone ASVs were also different between limestone and sandstone. For example, *Polyblastia* and *Verrucaria* in limestone correlated with ASVs belonging to the order Burkholderiales (*R* = 0.546, *P* = 0.018) and Rhizobiales (*R* = 0.545, *P* = 0.019), whereas *Porpidia* in sandstone correlated with genera *Singulisphaera* (*R* = 0.679, *P* = 0.001) and *Polymorphobacter* (*R* = 0.537, *P* = 0.021).

## Discussion

In this study, we showed that the community structure of four taxonomic groups (archaea, bacteria, fungi and protists) differed significantly between rocks and soils. Previous studies have demonstrated that the habitat heterogeneity has strong influences on lithic and edaphic microbial communities (Pointing et al., 2009; Lee et al., 2016; Meslier et al., 2018). For example, the diversity of microbial communities in four different lithic substrates from the Atacama Desert was found to be strongly correlated with those substrates (Meslier et al., 2018). Similarly, differences in diversity and structure of the microbial community have been reported for lithic and soil niches in Plateau Desert and McMurdo Dry Valleys (Pointing et al., 2009; Lee et al., 2016). To our knowledge, no study has compared community assembly across lithic and edaphic niches and without the confounding effect of different climate regimes in the high Arctic cold desert. We also presented for the first time a comprehensive multidomain diversity of lithic and edaphic microbial communities from Svalbard. Although lithic colonizations in Svalbard were previously reported, target taxa were mostly confined to bacteria and fungi.

A comprehensive assessment of the microbiome of rock and soil showed that Thaumarchaeota, Proteobacteria, Ascomycota, and Chlorophyta were the most abundant archaeal, bacterial, fungal, and protistan taxa, respectively. Thaumarchaeota, which is mostly composed of class Nitrososphaeria including a number of ammonia-oxidizing archaea (AOA), is globally distributed in aquatic and terrestrial ecosystems (Alves et al., 2018). AOA is especially known as a pioneer that can thrive in extreme and oligotrophic environments (Stahl and de la Torre, 2012; Alves et al., 2018). Their genetic flexibility and niche adaptability not only allow them to adapt to harsh environments but also potentially play a role as primary producers in the ecosystems, contributing to nitrogen cycling in nitrogen-limited ecosystems (Stahl and de la Torre, 2012; Beam et al., 2014). Proteobacteria are also known to contain a number of phototrophic, photoheterotrophic, and chemolithotrophic taxa that are able to survive in oligotrophic niches such as polar regions (Wei et al., 2016; Choe et al., 2018). For example, N-fixing Rhizobiales, Burkholderiales, Xanthomonadales, and Myxococcales belonging to the Proteobacteria have been reported to dominate in Arctic soils as well as in Antarctica (Cary et al., 2010; Kim et al., 2015; Malard and Pearce, 2018). Among the microbial eukaryotic groups, despite varying in relative abundance at lower taxonomic levels, the fungal phyla Ascomycota and the phyla Chlorophyta belonging to green algae dominated the study area. The Ascomycota contain a variety of taxonomic groups that can resist environmental stresses (Selbmann et al., 2005). For example, certain species of the class Lecanoromycetes and Leotiomycetes belonging to Ascomycota are able to produce fungal secondary metabolites such as atranorin, calycin, pinastric acid, and pulvinic acid that absorb strong UV and filter out excessive UV irradiation (Molnár and Farkas, 2010). Moreover, the ecological characteristics of various lichen-forming fungi, which comprise a large part of Ascomycota (about 40% of all Ascomycota), could be a possible reason for the predominance of these fungal phyla in the study area (Lawrey and Diederich, 2003). In this context, the high relative abundance of Trebouxiophyceae and Ulvophyceae belonging to Chlorophyta, which include symbiotic photobionts, parallels that of Ascomycota, and this correlation makes sense since the former includes symbiotic photobionts within lichens (Faluaburu et al., 2019).

Distinct differences were observed in the composition of microbial communities between lithic and edaphic niches. Similar results were also observed in previous studies by Pointing et al. (2009); Yung et al. (2014), Van Goethem et al. (2016), and Garrido-Benavent et al. (2020) which compared rock and soil microbial communities in Antarctic regions. In line with our findings, Garrido-Benavent et al. (2020) revealed differentially abundant bacterial and fungal taxa in rock and soil, showing that Deinococcus-Thermus and Lecanoromycetes were more abundant in lithic than edaphic communities, and the opposite was true for Agaricomycetes and Leotiomycetes. In contrast with our findings, Chloroflexi and Cyanobacteria showed low abundance in examined rock (igneous rock) in the study. These contrasting results are likely due to different rock types, supported by previous findings by Choe et al. (2018), which showed that Chloroflexi and Cyanobacteria were more abundant in sedimentary rocks than igneous rocks. This differential selection for taxa in the two niches is likely related to the capacity of the microorganisms to adapt to the niche and the biotic interactions between them. For example, bacterial phyla, Acidobacteria and Gemmatimonadetes were significantly higher in the edaphic niche than in the lithic niche. Additionally, Proteobacteria were identified as the most abundant edaphic specialist. These phyla are known to include aerobic anoxygenic phototrophic bacteria, which may play an important role in contributing to organic carbon cycling in nutrient-poor arid soils (Csotonyi et al., 2010). Furthermore, a previous study showed high proportions of Gemmatimonadetes in arid soils, suggesting an adaption to low-moisture environments (DeBruyn et al., 2011). As such, the functional similarity required to adapt to the extreme environment of the dominant taxa despite the different characteristics of the niche may be an example of functional redundancy (Louca et al., 2018). Functional redundancy has been observed in extreme environments such as deep subseafloor aquifer (Tully et al., 2018) and Antarctic terrestrial ecosystems (Yergeau et al., 2007, 2012; Pointing et al., 2009). For example, Pointing et al. (2009) showed little functional variation between the bare soil samples, despite the samples having heterogeneous phylogenetic diversity. Similarly, Yergeau et al. (2012) revealed that the major members of the soil community were functionally similar despite differences in microbial diversity between distinct habitats.

Lithic microbial communities are frequently dominated by Cyanobacteria, which are the major drivers of photosynthetic carbon fixation and nitrogen cycling in desert ecosystems (Warren-Rhodes et al., 2006; Chan et al., 2012). Consistent with these studies, our data showed that Cyanobacteria was significantly higher in the lithic niches than in edaphic niches. Particularly, ASVs belonging to the family Chroococcidiopsidaceae were significantly higher in both limestone and sandstone than in soils. Among the species belonging to Chroococcidiopsidaceae, Chroococcidiopsis, which are most commonly observed in the lithic niche, have relatively small coccoid cells. It is well adapted to entering pore spaces in the rock matrix as well as secreting extracellular polymeric substances that are implicated in various stress resistances in rocks (Chan et al., 2012; Pointing, 2016). These characteristics could be a possible reason for the predominance of the cyanobacterial family in the lithic niches. The relative abundances of Actinobacteria, FBP, and Deinococcus-Thermus were also higher in the lithic communities. The order Solirubrobacterales in Actinobacteria were the most abundant group observed in lithic niches, consistent with previous studies that have been observed in rocks (Esposito et al., 2015; Khilyas et al., 2019). The 16S rRNA sequence of candidate division FBP was first reported from a lichen-dominated lithic habitat in Antarctica (de la Torre et al., 2003), and Deinococcus-Thermus was also dominant in cryptoendolithic environments (Cary et al., 2010). The prominent features of these phyla are that they can be adapted to various environmental stresses, such as fluctuating temperature and strong UV radiation (Suzuki and Whitman, 2012). Such stress tolerance traits can help them survive in lithic habitats in polar regions.

Fungal ASVs belonging to Lecanoromycetes and Eurotiomycetes were notably higher in both limestone and sandstone than in soils and were also the most abundant specialists in the lithic niche. The majority of the Lecanoromycetes and Eurotiomycetes were identified as members of the lichen-forming order Lecanorales and Verrucariales. Lichens are considered especially well adapted to the lithic niche under cold environments, owing to their freezing tolerance, low mineral nutrient demand, and ability to be photosynthetically active at low temperatures (Kappen, 2000). Consistent with our findings, a recent study on lithic colonization patterns in Axel Heiberg Island, Inglefield Land, and Svalbard has reported a lichen mycobiont prevalence in lithic niches (Hansen et al., 2006; Ziolkowski et al., 2013; Choe et al., 2018). Unlike lithic niches, the relative abundance of ASVs belonging to class Leotiomycetes was not only higher in the edaphic niche than in the lithic niche, but was also identified as a specialist in the soils. The dominant fungal orders detected in this study belonged to Helotiales (Leotiomycetes) in Ascomycota. In previous studies, Helotiales was found in the rhizosphere in Arctic regions (Bjorbaekmo et al., 2010; Walker et al., 2011). Deslippe et al. (2012) observed that Ascomycota was dominated by Helotiales in Alaska (low Arctic). In the Siberian tundra (High Arctic), Ascomycota was also dominated by Leotimycetes (Gittel et al., 2014). In this respect, Leotimycetes seem to be more optimized or preferred for the edaphic niche.

The classes Trebouxiophyceae and Ulvophyceae in Chlorophyta were notably higher in both limestone and sandstone than in soils. According to previous studies, Cyanobacteria and Chlorophyta (green algae) are regarded as the pioneering inhabitants in the colonization of the lithic niche (Tiano et al., 1995). Their biofilm-forming and photosynthetic ability allow them to colonize on a rock by developing a symbiotic relationship with lichen-forming fungal taxa (de los Rios et al., 2007). The higher relative abundances of Sarcomonadea and Spirotrichea in soils were in line with previous soil eukaryotic DNA studies as well as microscopic studies (Adl and Gupta, 2006; Bates et al., 2013; Harder et al., 2016; Seppey et al., 2017). Cercozoa, to which Sarcomonadea belongs, tended to be more abundant in arid soils, and ciliate taxa such as Spirotrichea are known to survive in arid conditions such as polar deserts (Adl and Gupta, 2006; Bates et al., 2013). The distinct communities between rocks and soils demonstrate that distinct microbial communities can be induced by different niche characteristics, even though they are close to each other.

Interestingly, our results provide evidence for ecological coherence at higher taxonomic ranks in lithic bacterial and fungal communities. The lithic niches were dominated by similar phyla that were clearly distinguished from those of the edaphic niches, but the composition of lithic communities at lower taxonomic levels differed by rock type. This ecological coherence depending on niche implies that its members share life strategies or specific traits that distinguish them from other taxa, but the functional traits of those communities need further investigation (Philippot et al., 2010). In other words, the spatial heterogeneity of the lithic microhabitats likely exerts a stronger filter on the ability of airborne microorganisms to colonize the lithic substrate compared to the edaphic substrate in the stochastic process of dispersal, which is consistent with the results in previous studies (de los Rios et al., 2007; Meslier et al., 2018).

Rocks provide habitats for microorganisms, and they also affect the microbial community structure (Meslier et al., 2018). Although Meslier et al. (2018) suggested that the chemical properties of the rock may not be an essential driver of community composition and diversity, the ability of microorganisms to use nutrients leached from rocks can affect the community composition in their niche. In addition, Cámara et al. (2008) suggested that the microstructure of the rock, such as the space available for colonization and its physical structure, linked to water retention capabilities, can be a determinant of the potential bioreceptivity of lithic habitat. In our study, despite the clearly different chemical composition and microhabitat architecture of limestone and sandstone, the structures of archaeal, bacterial, and protistan communities were similar in both limestone and sandstone, which is consistent with the previous studies on lithic communities from polar deserts (de la Torre et al., 2003; Pointing et al., 2007; Archer et al., 2017). This suggests that the dominant microbial taxa of these communities consist of common microorganisms that can colonize lithic microhabitats, supporting the microbial meta-community concept (Walker and Pace, 2007) and also imply that the climatic characteristics of the habitat could be major drivers determining the dominant taxa in lithic niches (Pointing et al., 2009; Cowan et al., 2010).

Nevertheless, our results revealed obvious differences that were mainly caused by lichen-forming fungal taxa in the fungal community structure between limestone and sandstone. Such differences were not only observed in relative abundance at the low taxonomic level between limestone and sandstone but the network analysis also showed that different fungal genera are keystone taxa in limestone and sandstone. The fungal ASVs identified as keystone taxa in limestone were mostly assigned to *Polyblastia* and *Verrucaria* belonging to Verrucariales. Members of these genera are well known to form calcicolous endolithic lichens (Halda, 2003; Hoppert et al., 2004). In contrast to limestone, fungal ASVs belonging to *Porpidia* and *Candelariella* were identified as keystone taxa in sandstone. They are frequently observed in siliceous rocks such as sandstone (Dobson, 1979). Although biophysical and biochemical mechanisms of rock weathering by lichens have been extensively studied (Mccarroll and Viles, 1995; Matthews and Owen, 2008; de los Rios et al., 2009; Favero-Longo et al., 2011), evolutionary links between habitat preference and lichen-forming fungal taxa have been rarely investigated (Hoppert et al., 2004). One possible reason for this could be that bioreceptivity of rocks is determined by chemical, mineralogical, and physical properties such as porosity, pore size, pore distribution, pore connectivity, water retention capacity, and light transmittance (Wierzchos et al., 2015). Previous studies showed that the differential rock colonization by lichen-associated fungi is largely determined by differences in the physicochemical characteristics of the lithic substrate (Kurtz and Netoff, 2001; Hoppert et al., 2004; Favero-Longo et al., 2011). Furthermore, lichenized fungi play a pivotal role in the production of extracellular compounds as well as in the formation of lichen thallus structures (Lawrey and Diederich, 2003). In this context, it is not surprising that lichen-associated fungi might act as hubs in the community network, and could be habitat specialized taxa depending on rock type.

## Conclusion

Niche differentiation of the microbiomes in lithic and edaphic substrates in this study was demonstrated using the multidomain diversity assessment by sampling strategy that minimized the effect of biogeography. The different physical and chemical properties of lithic and edaphic niches supported the distinct microbial communities, even in close spatial proximity. In particular, for lithic niches, we found that lichen-forming fungi played key roles as a hub in the communities in the cold desert. Taken together, this study provides insights into the microbial ecology of lithic and edaphic niches that influence microbial community composition in a high arctic polar desert. To comprehensively understand the mechanisms of microbial community assembly, ongoing work examining their functional diversity and environmental factors at the micro-scale will help understand how the microbial assemblages adapt to their niches in the cold desert.

## Data Availability Statement

The datasets generated for this study can be found in the Raw sequencing data were deposited in the Sequence Read Archive (SRA) of NCBI under the BioProject ID PRJNA380676.

## Author Contributions

Y-HC conceived the setup, did rock and soil sample fieldwork, conducted the microbial lab work, analyzed the data, and wrote the manuscript. MK provided a range of feedback on the analyses and the manuscript. YL supervised the setup and provided funding. All authors reviewed and contributed to the final version of the manuscript.

## Conflict of Interest

The authors declare that the research was conducted in the absence of any commercial or financial relationships that could be construed as a potential conflict of interest.

## Footnotes

1 https://www.bioinfor-matics.babraham.ac.uk/projects/fastqc/

2 https://qiime2.org/

3 https://github.com/qiime2/q2-feature-classifier

4 https://cran.r-project.org/web/packages/vegan/index.html
