# Supplementary material for: Distinct Microbial Communities in Adjacent Rock and Soil Substrates on a High Arctic Polar Desert
Source: Front Microbiol. 2021 Jan 8;11:607396. doi: 10.3389/fmicb.2020.607396 (PMC7819959; doi:10.3389/fmicb.2020.607396)
Supplement: Supplementary file 1 [file Data_Sheet_1.docx]

**1. Supplementary Figures and Tables**

**
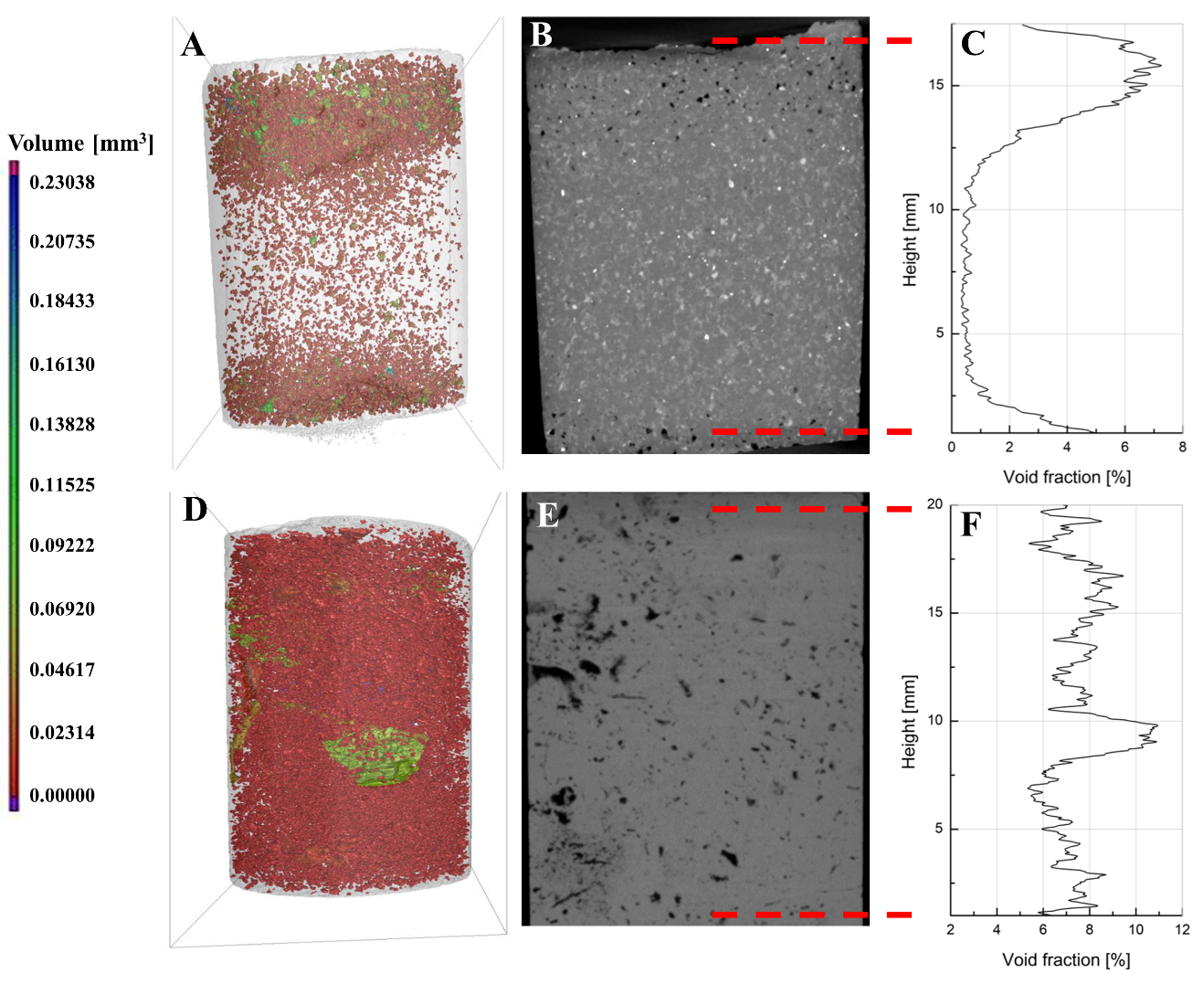
**

**Figure S1.** Result of X-ray CT in sandstone (A-C) and limestone (D-F). Spatial distribution of pores (A, D); pores in longitudinal section (B, E); porosity change along with the height (C, F)

**
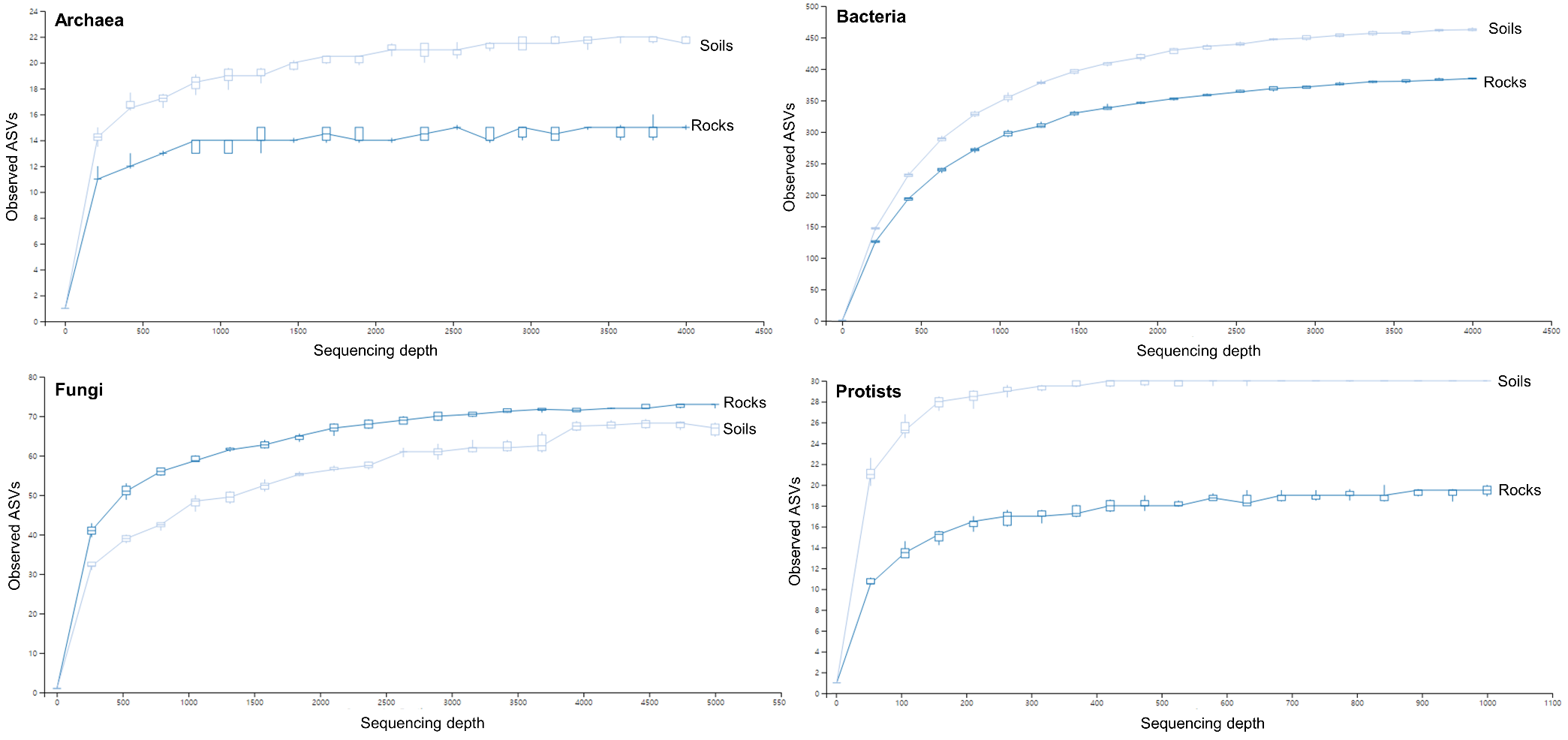
**

**Figure S2.** Rarefaction curves for archaeal, bacterial, fungal and protistan ASVs data and considering niche type (rocks and soils)


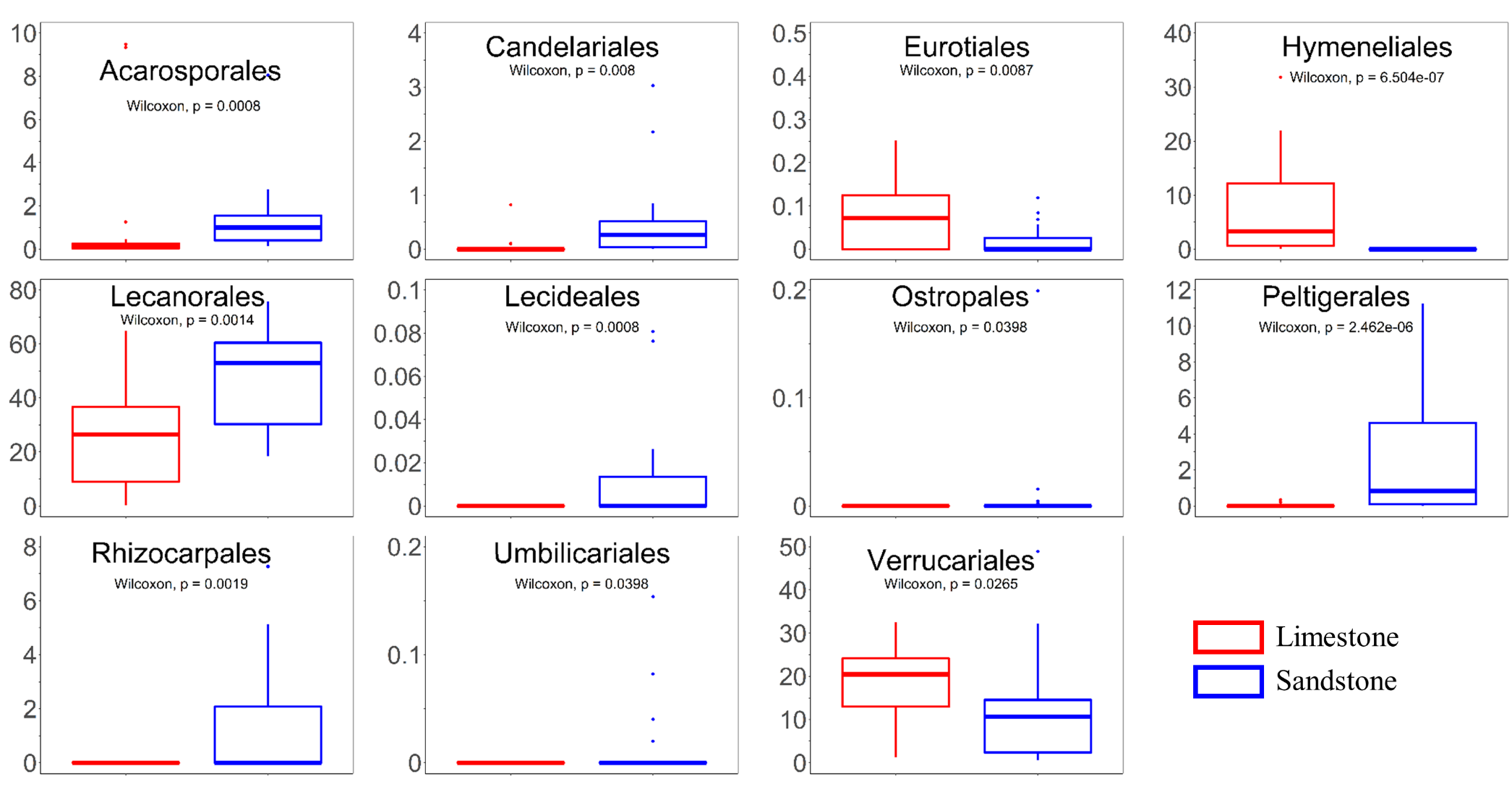


**Figure S3.** The relative abundance of fungal taxa at order level in limestones and sandstones


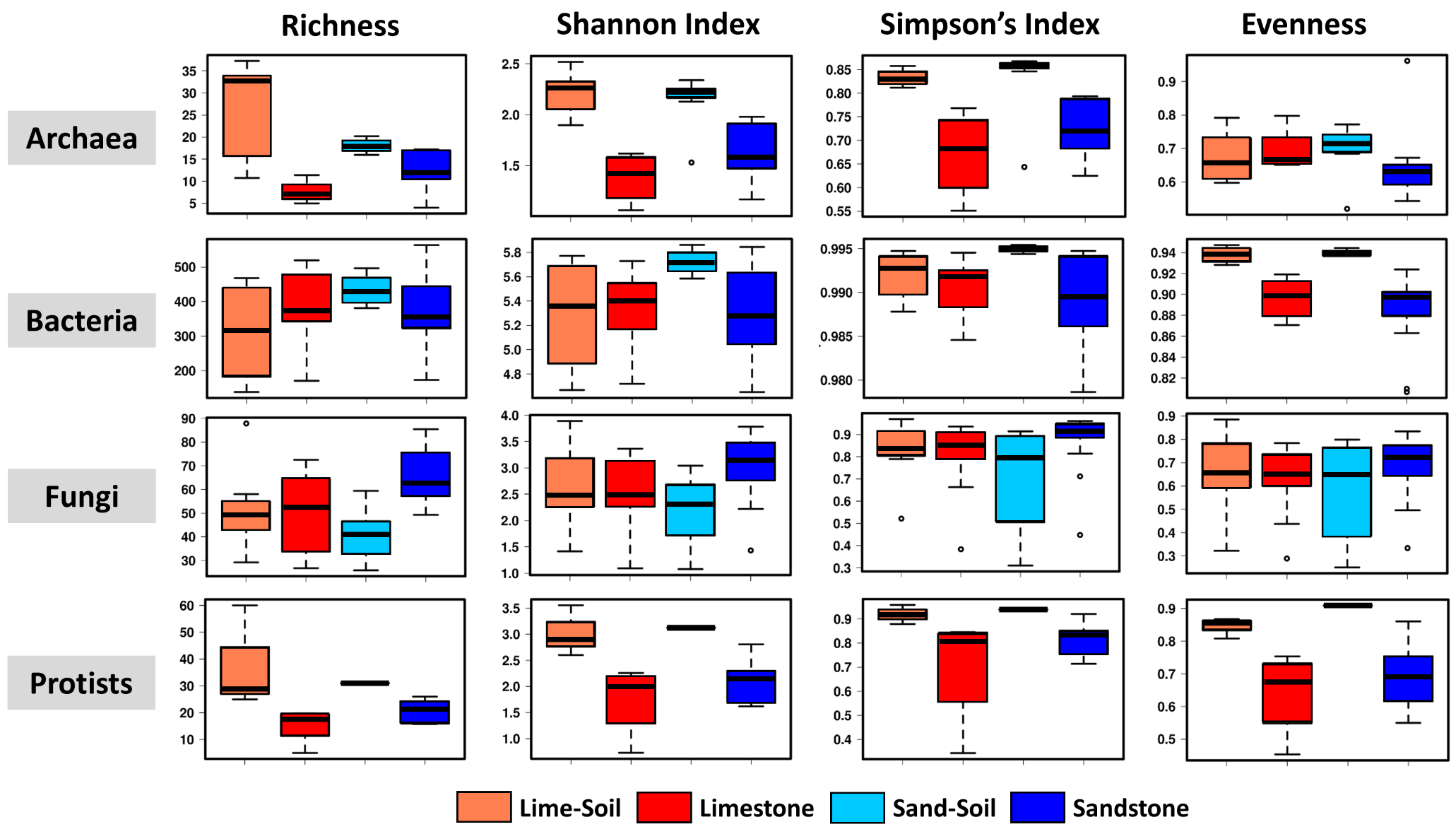


**Figure S4.** Alpha-diversity estimators for four organism groups according to niche type (rocks and soils)


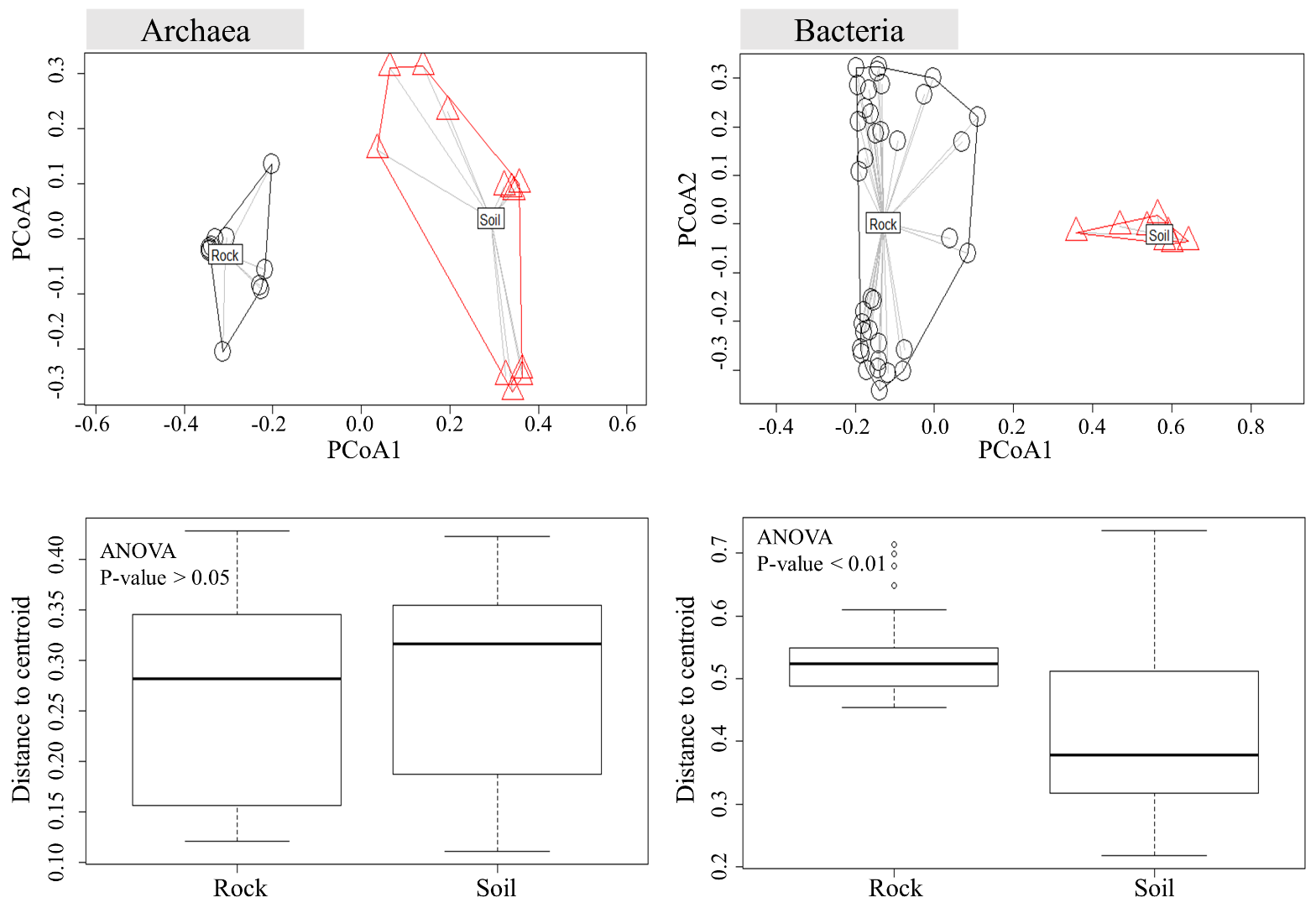


**Figure S5.** Principal Coordinates Analysis (PCoA) plots of Bray-Curtis dissimilarities representing the results of multivariate homogeneity tests that compare sample dispersion between rocks and soils in archaeal and bacteria communities. Boxplots in the lower panel illustrate summary data related to distances to centroids.


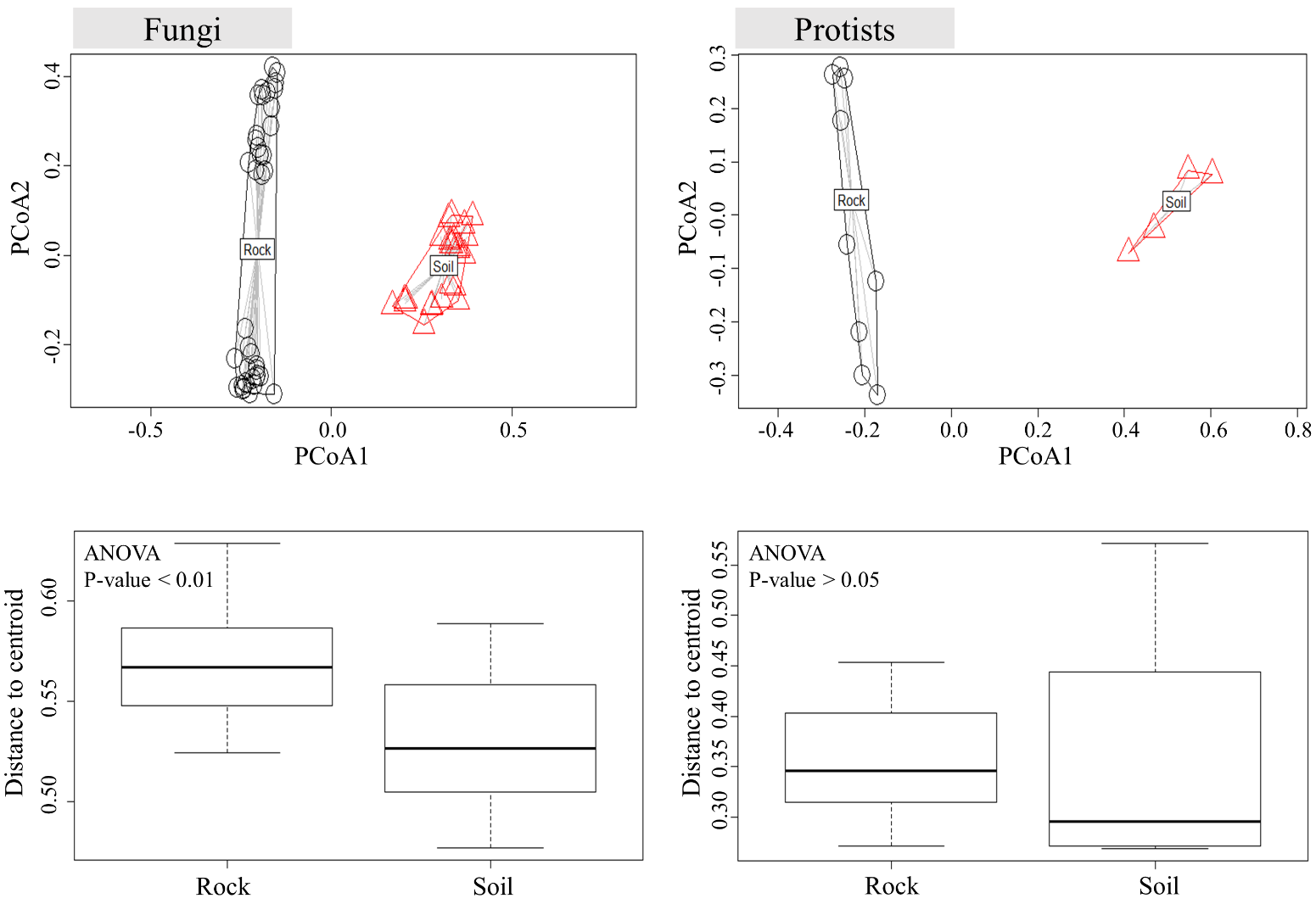


**Figure S6.** Principal Coordinates Analysis (PCoA) plots of Bray-Curtis dissimilarities representing the results of multivariate homogeneity tests that compare sample dispersion between rocks and soils in fungal and protistan communities. Boxplots in the lower panel illustrate summary data related to distances to centroids.

**Table S1** List of all samples in this study

| Sample | Type | GPS coordinates | Sampling data | Archaea | Bacteria | Fungi | Eukaryotes |
| --- | --- | --- | --- | --- | --- | --- | --- |
| Lime1 | Limestone | N 78º55.932' E 011º46.270' | 10. Aug. 2016 |  |  |  |  |
| Lime2 | Limestone | N 78º55.932' E 011º46.270' | 10. Aug. 2016 |  |  |  |  |
| Lime3 | Limestone | N 78º55.932' E 011º46.270' | 10. Aug. 2016 |  |  |  |  |
| Lime4 | Limestone | N 78º55.932' E 011º46.270' | 10. Aug. 2016 |  |  |  |  |
| Lime5 | Limestone | N 78º55.932' E 011º46.270' | 10. Aug. 2016 |  |  |  |  |
| Lime6 | Limestone | N 78º55.731' E 011º46.371' | 10. Aug. 2016 |  |  |  |  |
| Lime7 | Limestone | N 78º55.731' E 011º46.371' | 10. Aug. 2016 |  |  |  |  |
| Lime8 | Limestone | N 78º55.731' E 011º46.371' | 10. Aug. 2016 |  |  |  |  |
| Lime9 | Limestone | N 78º55.731' E 011º46.371' | 10. Aug. 2016 |  |  |  |  |
| Lime10 | Limestone | N 78º55.531' E 011º46.771' | 10. Aug. 2016 |  |  |  |  |
| Lime11 | Limestone | N 78º55.531' E 011º46.771' | 10. Aug. 2016 |  |  |  |  |
| Lime12 | Limestone | N 78º55.531' E 011º46.771' | 10. Aug. 2016 |  |  |  |  |
| Lime13 | Limestone | N 78º55.531' E 011º46.771' | 10. Aug. 2016 |  |  |  |  |
| Lime14 | Limestone | N 78º55.531' E 011º46.771' | 10. Aug. 2016 |  |  |  |  |
| Lime15 | Limestone | N 78º55.231' E 011º46.871' | 10. Aug. 2016 |  |  |  |  |
| Lime16 | Limestone | N 78º55.231' E 011º46.871' | 10. Aug. 2016 |  |  |  |  |
| Lime17 | Limestone | N 78º55.231' E 011º46.871' | 10. Aug. 2016 |  |  |  |  |
| Lime18 | Limestone | N 78º55.231' E 011º46.871' | 10. Aug. 2016 |  |  |  |  |
| Lime19 | Limestone | N 78º55.231' E 011º46.871' | 10. Aug. 2016 |  |  |  |  |
| Lime-Soil1 | Soil | N 78º55.932' E 011º46.270' | 10. Aug. 2016 |  |  |  |  |
| Lime-Soil2 | Soil | N 78º55.932' E 011º46.270' | 10. Aug. 2016 |  |  |  |  |
| Lime-Soil3 | Soil | N 78º55.932' E 011º46.270' | 10. Aug. 2016 |  |  |  |  |
| Lime-Soil4 | Soil | N 78º55.932' E 011º46.270' | 10. Aug. 2016 |  |  |  |  |
| Lime-Soil5 | Soil | N 78º55.932' E 011º46.270' | 10. Aug. 2016 |  |  |  |  |
| Lime-Soil6 | Soil | N 78º55.731' E 011º46.371' | 10. Aug. 2016 |  |  |  |  |
| Lime-Soil7 | Soil | N 78º55.731' E 011º46.371' | 10. Aug. 2016 |  |  |  |  |
| Lime-Soil8 | Soil | N 78º55.731' E 011º46.371' | 10. Aug. 2016 |  |  |  |  |
| Lime-Soil9 | Soil | N 78º55.731' E 011º46.371' | 10. Aug. 2016 |  |  |  |  |
| Lime-Soil10 | Soil | N 78º55.531' E 011º46.771' | 10. Aug. 2016 |  |  |  |  |
| Lime-Soil11 | Soil | N 78º55.531' E 011º46.771' | 10. Aug. 2016 |  |  |  |  |
| Lime-Soil12 | Soil | N 78º55.531' E 011º46.771' | 10. Aug. 2016 |  |  |  |  |
| Lime-Soil13 | Soil | N 78º55.531' E 011º46.771' | 10. Aug. 2016 |  |  |  |  |
| Lime-Soil14 | Soil | N 78º55.531' E 011º46.771' | 10. Aug. 2016 |  |  |  |  |
| Lime-Soil15 | Soil | N 78º55.231' E 011º46.871' | 10. Aug. 2016 |  |  |  |  |
| Lime-Soil16 | Soil | N 78º55.231' E 011º46.871' | 10. Aug. 2016 |  |  |  |  |
| Lime-Soil17 | Soil | N 78º55.231' E 011º46.871' | 10. Aug. 2016 |  |  |  |  |
| Lime-Soil18 | Soil | N 78º55.231' E 011º46.871' | 10. Aug. 2016 |  |  |  |  |
| Lime-Soil19 | Soil | N 78º55.231' E 011º46.871' | 10. Aug. 2016 |  |  |  |  |
| Sand1 | Sandstone | N 78º55.866' E 011º46.407' | 16. Aug. 2016 |  |  |  |  |
| Sand2 | Sandstone | N 78º55.866' E 011º46.407' | 16. Aug. 2016 |  |  |  |  |
| Sand3 | Sandstone | N 78º55.866' E 011º46.407' | 16. Aug. 2016 |  |  |  |  |
| Sand4 | Sandstone | 78º55.868' E 011º46.432' | 16. Aug. 2016 |  |  |  |  |
| Sand5 | Sandstone | 78º55.868' E 011º46.432' | 16. Aug. 2016 |  |  |  |  |
| Sand6 | Sandstone | 78º55.868' E 011º46.432' | 16. Aug. 2016 |  |  |  |  |
| Sand7 | Sandstone | N 78º55.944' E 011º46.193' | 16. Aug. 2016 |  |  |  |  |
| Sand8 | Sandstone | N 78º55.944' E 011º46.193' | 16. Aug. 2016 |  |  |  |  |
| Sand9 | Sandstone | N 78º55.944' E 011º46.193' | 16. Aug. 2016 |  |  |  |  |
| Sand10 | Sandstone | N 78º56.011' E 011º45.901' | 16. Aug. 2016 |  |  |  |  |
| Sand11 | Sandstone | N 78º56.011' E 011º45.901' | 16. Aug. 2016 |  |  |  |  |
| Sand12 | Sandstone | N 78º56.011' E 011º45.901' | 16. Aug. 2016 |  |  |  |  |
| Sand13 | Sandstone | N 78º56.011' E 011º45.901' | 16. Aug. 2016 |  |  |  |  |
| Sand14 | Sandstone | N 78º55.953' E 011º46.071' | 16. Aug. 2016 |  |  |  |  |
| Sand15 | Sandstone | N 78º55.953' E 011º46.071' | 16. Aug. 2016 |  |  |  |  |
| Sand16 | Sandstone | N 78º55.953' E 011º46.071' | 16. Aug. 2016 |  |  |  |  |
| Sand17 | Sandstone | N 78º55.922' E 011º46.292' | 16. Aug. 2016 |  |  |  |  |
| Sand18 | Sandstone | N 78º55.922' E 011º46.292' | 16. Aug. 2016 |  |  |  |  |
| Sand19 | Sandstone | N 78º55.922' E 011º46.292' | 16. Aug. 2016 |  |  |  |  |
| Sand-Soil1 | Soil | N 78º55.866' E 011º46.407' | 16. Aug. 2016 |  |  |  |  |
| Sand-Soil2 | Soil | N 78º55.866' E 011º46.407' | 16. Aug. 2016 |  |  |  |  |
| Sand-Soil3 | Soil | N 78º55.866' E 011º46.407' | 16. Aug. 2016 |  |  |  |  |
| Sand-Soil4 | Soil | 78º55.868' E 011º46.432' | 16. Aug. 2016 |  |  |  |  |
| Sand-Soil5 | Soil | 78º55.868' E 011º46.432' | 16. Aug. 2016 |  |  |  |  |
| Sand-Soil6 | Soil | 78º55.868' E 011º46.432' | 16. Aug. 2016 |  |  |  |  |
| Sand-Soil7 | Soil | N 78º55.944' E 011º46.193' | 16. Aug. 2016 |  |  |  |  |
| Sand-Soil8 | Soil | N 78º55.944' E 011º46.193' | 16. Aug. 2016 |  |  |  |  |
| Sand-Soil9 | Soil | N 78º55.944' E 011º46.193' | 16. Aug. 2016 |  |  |  |  |
| Sand-Soil10 | Soil | N 78º56.011' E 011º45.901' | 16. Aug. 2016 |  |  |  |  |
| Sand-Soil11 | Soil | N 78º56.011' E 011º45.901' | 16. Aug. 2016 |  |  |  |  |
| Sand-Soil12 | Soil | N 78º56.011' E 011º45.901' | 16. Aug. 2016 |  |  |  |  |
| Sand-Soil13 | Soil | N 78º56.011' E 011º45.901' | 16. Aug. 2016 |  |  |  |  |
| Sand-Soil14 | Soil | N 78º55.953' E 011º46.071' | 16. Aug. 2016 |  |  |  |  |
| Sand-Soil15 | Soil | N 78º55.953' E 011º46.071' | 16. Aug. 2016 |  |  |  |  |
| Sand-Soil16 | Soil | N 78º55.953' E 011º46.071' | 16. Aug. 2016 |  |  |  |  |
| Sand-Soil17 | Soil | N 78º55.922' E 011º46.292' | 16. Aug. 2016 |  |  |  |  |
| Sand-Soil18 | Soil | N 78º55.922' E 011º46.292' | 16. Aug. 2016 |  |  |  |  |
| Sand-Soil19 | Soil | N 78º55.922' E 011º46.292' | 16. Aug. 2016 |  |  |  |  |

The green boxes indicate samples that succeeded in amplification or DNA sequencing. Red boxes indicate samples that failed amplification or DNA sequencing.

**Table S2** Summary information for the obtained sequence data from four organism groups

| Organism group | Sample | Input sequences | Filtered sequences | Denoised sequences | Merged sequences | Non-chimeric sequences |
| --- | --- | --- | --- | --- | --- | --- |
| Archaea | A-L1 | 6402 | 5515 | 5486 | 4830 | 4767 |
|  | A-L2 | 12432 | 2282 | 2229 | 2206 | 2158 |
|  | A-LS13 | 13424 | 12853 | 12742 | 12557 | 11898 |
|  | A-LS16 | 33274 | 31825 | 31639 | 31232 | 28780 |
|  | A-LS17 | 39638 | 37691 | 37299 | 36487 | 32837 |
|  | A-LS18 | 35979 | 34342 | 34051 | 33423 | 30322 |
|  | A-LS19 | 31984 | 30343 | 30030 | 29570 | 27972 |
|  | A-S1 | 22154 | 1565 | 1486 | 1468 | 1439 |
|  | A-S3 | 29222 | 6022 | 5917 | 5669 | 5308 |
|  | A-S4 | 22499 | 21421 | 21328 | 21186 | 20903 |
|  | A-S5 | 28298 | 26932 | 26723 | 26470 | 26081 |
|  | A-S6 | 25865 | 24282 | 24107 | 23682 | 23409 |
|  | A-S16 | 35277 | 33434 | 33323 | 32544 | 31174 |
|  | A-S17 | 43453 | 40904 | 40790 | 39562 | 38071 |
|  | A-S18 | 33731 | 31662 | 31574 | 30643 | 29454 |
|  | A-S19 | 23515 | 22163 | 22103 | 21502 | 20898 |
|  | A-SS5 | 10452 | 9966 | 9904 | 9702 | 9247 |
|  | A-SS8 | 30010 | 28770 | 28660 | 28345 | 27465 |
|  | A-SS9 | 26374 | 25066 | 24913 | 24522 | 23606 |
|  | A-SS10 | 23539 | 22478 | 22363 | 22048 | 21516 |
|  | A-SS11 | 27193 | 25907 | 25796 | 25415 | 24742 |
|  | A-SS12 | 9544 | 8875 | 8785 | 8299 | 7912 |
|  | A-SS13 | 7790 | 7289 | 7237 | 6774 | 6477 |
| Bacteria | B-L1 | 15266 | 13325 | 11719 | 6783 | 6161 |
|  | B-L2 | 18593 | 16774 | 14221 | 7157 | 6346 |
|  | B-L3 | 39602 | 34288 | 30383 | 16908 | 15658 |
|  | B-L4 | 41445 | 37650 | 36592 | 32128 | 31481 |
|  | B-L5 | 19376 | 17440 | 16978 | 15666 | 15433 |
|  | B-L6 | 27161 | 24479 | 23963 | 22568 | 22214 |
|  | B-L7 | 27075 | 24323 | 23846 | 21679 | 21379 |
|  | B-L8 | 14098 | 12660 | 12423 | 11346 | 11162 |
|  | B-L9 | 24837 | 21981 | 21543 | 20243 | 20101 |
|  | B-L10 | 27411 | 24757 | 23583 | 18467 | 17884 |
|  | B-L12 | 28981 | 26242 | 25545 | 23221 | 22884 |
|  | B-L13 | 22439 | 20333 | 19808 | 18086 | 17871 |
|  | B-L14 | 17601 | 15910 | 15289 | 13639 | 13424 |
|  | B-L15 | 28007 | 25122 | 24593 | 22558 | 22311 |
|  | B-L16 | 46842 | 42343 | 41567 | 38724 | 38075 |
|  | B-L17 | 34501 | 31152 | 30447 | 28278 | 27754 |
|  | B-L18 | 35266 | 31834 | 30952 | 28251 | 27511 |
|  | B-L19 | 35126 | 31763 | 30975 | 28041 | 27521 |
|  | B-LS16 | 15563 | 12106 | 9781 | 4545 | 4443 |
|  | B-LS17 | 24537 | 22408 | 20326 | 16326 | 15964 |
|  | B-LS18 | 28986 | 26513 | 23990 | 18619 | 18164 |
|  | B-LS19 | 10378 | 9484 | 8084 | 6216 | 6056 |
|  | B-S1 | 91383 | 78492 | 72186 | 45187 | 40404 |
|  | B-S2 | 79086 | 68847 | 65147 | 47043 | 40606 |
|  | B-S3 | 60134 | 51611 | 46694 | 29824 | 26386 |
|  | B-S4 | 26451 | 24213 | 23138 | 20724 | 20356 |
|  | B-S5 | 28705 | 26202 | 25204 | 22169 | 21577 |
|  | B-S6 | 47309 | 42987 | 41683 | 35729 | 34481 |
|  | B-S7 | 40806 | 37145 | 35941 | 32020 | 31191 |
|  | B-S8 | 51865 | 47136 | 46074 | 40774 | 39064 |
|  | B-S9 | 49584 | 45407 | 44922 | 43498 | 42773 |
|  | B-S10 | 55117 | 50173 | 49513 | 46538 | 44922 |
|  | B-S11 | 24065 | 21801 | 21395 | 20309 | 20197 |
|  | B-S12 | 20939 | 19081 | 18440 | 16911 | 16686 |
|  | B-S13 | 10091 | 9167 | 8721 | 7660 | 7571 |
|  | B-S14 | 24025 | 21916 | 21303 | 19047 | 18764 |
|  | B-S15 | 17222 | 15632 | 15090 | 13660 | 13418 |
|  | B-S16 | 23146 | 21093 | 20172 | 18072 | 17652 |
|  | B-S17 | 7668 | 6947 | 6426 | 5476 | 5258 |
|  | B-S18 | 14543 | 13258 | 12623 | 10945 | 10652 |
|  | B-SS8 | 30695 | 27694 | 24978 | 18300 | 17559 |
|  | B-SS9 | 23226 | 20881 | 18792 | 14095 | 13667 |
|  | B-SS10 | 35138 | 32054 | 28817 | 20511 | 19779 |
|  | B-SS11 | 20106 | 18275 | 16204 | 12231 | 11966 |
| Fungi | F-L1 | 92650 | 38263 | 37586 | 34542 | 32251 |
|  | F-L2 | 103536 | 50918 | 50211 | 47745 | 43398 |
|  | F-L3 | 60790 | 28954 | 28425 | 25915 | 24169 |
|  | F-L4 | 56885 | 16629 | 16363 | 15688 | 15225 |
|  | F-L5 | 41812 | 12431 | 12270 | 12060 | 11961 |
|  | F-L6 | 24950 | 8914 | 8728 | 8429 | 8359 |
|  | F-L7 | 27071 | 11264 | 11131 | 10890 | 10825 |
|  | F-L8 | 8529 | 4084 | 3956 | 3915 | 3915 |
|  | F-L9 | 35627 | 11017 | 10869 | 10417 | 10351 |
|  | F-L10 | 33033 | 11892 | 11701 | 11054 | 10492 |
|  | F-L11 | 36985 | 23091 | 22898 | 22629 | 22484 |
|  | F-L12 | 24558 | 9098 | 8878 | 8597 | 8584 |
|  | F-L13 | 26914 | 11886 | 11695 | 11130 | 11091 |
|  | F-L14 | 27944 | 13149 | 12901 | 12616 | 12550 |
|  | F-L15 | 7065 | 2746 | 2636 | 2543 | 2529 |
|  | F-L16 | 25463 | 16748 | 16573 | 16087 | 16031 |
|  | F-L17 | 32931 | 23447 | 23253 | 21940 | 21665 |
|  | F-L18 | 28675 | 19139 | 18924 | 18304 | 18178 |
|  | F-L19 | 24525 | 15767 | 15602 | 14890 | 14820 |
|  | F-LS8 | 13980 | 1941 | 1749 | 1517 | 1515 |
|  | F-LS9 | 10032 | 2856 | 2635 | 2366 | 2362 |
|  | F-LS10 | 13578 | 4494 | 4269 | 3958 | 3944 |
|  | F-LS11 | 16108 | 4266 | 4055 | 3607 | 3591 |
|  | F-LS13 | 14142 | 4530 | 4309 | 4123 | 4071 |
|  | F-LS14 | 9411 | 3155 | 2935 | 2782 | 2768 |
|  | F-LS15 | 12662 | 6688 | 6507 | 6371 | 6306 |
|  | F-LS16 | 49772 | 11381 | 10882 | 9894 | 9858 |
|  | F-LS17 | 36379 | 16136 | 15598 | 14761 | 14565 |
|  | F-LS18 | 48573 | 21988 | 21380 | 20958 | 20753 |
|  | F-LS19 | 74153 | 4924 | 4598 | 3706 | 3389 |
|  | F-S1 | 93723 | 35272 | 34807 | 32999 | 31942 |
|  | F-S2 | 84720 | 45462 | 44757 | 42584 | 39569 |
|  | F-S3 | 109225 | 24243 | 23608 | 22174 | 20464 |
|  | F-S4 | 57145 | 17245 | 16748 | 16050 | 15945 |
|  | F-S5 | 83430 | 52237 | 51636 | 50418 | 47274 |
|  | F-S6 | 95940 | 26954 | 26647 | 25969 | 25374 |
|  | F-S7 | 67115 | 25296 | 24845 | 23976 | 23568 |
|  | F-S8 | 78132 | 39886 | 39432 | 38586 | 36199 |
|  | F-S9 | 112387 | 35392 | 35098 | 34174 | 32601 |
|  | F-S10 | 100063 | 34710 | 34344 | 33767 | 31709 |
|  | F-S11 | 89336 | 35334 | 34836 | 33993 | 32693 |
|  | F-S12 | 72846 | 19200 | 18716 | 17736 | 17214 |
|  | F-S13 | 78540 | 15121 | 14720 | 13850 | 13404 |
|  | F-S14 | 75956 | 22205 | 21607 | 20876 | 20255 |
|  | F-S15 | 96948 | 16575 | 16076 | 15310 | 14767 |
|  | F-S16 | 89814 | 20440 | 20046 | 19286 | 17372 |
|  | F-S17 | 86290 | 25179 | 24771 | 24100 | 22522 |
|  | F-S18 | 74578 | 34698 | 34265 | 33508 | 31773 |
|  | F-S19 | 53885 | 13495 | 13154 | 12509 | 11903 |
|  | F-SS4 | 7341 | 1681 | 1484 | 1399 | 1399 |
|  | F-SS5 | 11660 | 4339 | 4105 | 3882 | 3851 |
|  | F-SS6 | 13644 | 4692 | 4458 | 4153 | 4126 |
|  | F-SS7 | 13262 | 2900 | 2664 | 2464 | 2459 |
|  | F-SS8 | 35781 | 20565 | 20362 | 20063 | 19889 |
|  | F-SS9 | 57603 | 41084 | 40587 | 40123 | 39847 |
|  | F-SS10 | 41445 | 25296 | 24988 | 24413 | 24193 |
|  | F-SS11 | 22101 | 11626 | 11325 | 10767 | 10751 |
|  | F-SS12 | 5071 | 1548 | 1429 | 1244 | 1244 |
|  | F-SS13 | 4501 | 1487 | 1353 | 1246 | 1244 |
|  | F-SS14 | 5345 | 3986 | 3986 | 3746 | 3746 |
|  | F-SS15 | 5439 | 1976 | 1872 | 1735 | 1728 |
|  | F-SS17 | 7942 | 3481 | 3275 | 2970 | 2970 |
|  | F-SS18 | 6681 | 5044 | 5044 | 4873 | 4873 |
| Protists | P-L1 | 82007 | 68242 | 67385 | 57733 | 32530 |
|  | P-L2 | 71139 | 59156 | 58255 | 46629 | 26432 |
|  | P-L3 | 19882 | 16174 | 15967 | 13650 | 9883 |
|  | P-LS32 | 5333 | 4801 | 4102 | 3027 | 3017 |
|  | P-LS33 | 24246 | 22797 | 21848 | 18151 | 17941 |
|  | P-LS35 | 13398 | 12337 | 11755 | 10678 | 10600 |
|  | P-S1 | 32377 | 26488 | 26225 | 22983 | 19307 |
|  | P-S2 | 48451 | 40340 | 39958 | 35899 | 29418 |
|  | P-S3 | 44468 | 36914 | 36546 | 30646 | 22155 |
|  | P-S12 | 27437 | 25649 | 25479 | 22810 | 22094 |
|  | P-S14 | 22803 | 21199 | 21052 | 17979 | 17852 |
|  | P-S15 | 15965 | 14931 | 14794 | 13584 | 13164 |
|  | P-SS10 | 21110 | 18338 | 18005 | 15156 | 14997 |

**Table S3** Major and trace element compositions of selected samples (n=3)

|  | Limestone1 | Limestone2 | Limestone3 | Sandstone1 | Sandstone2 | Sandstone3 | Lime-Soil1 | Lime-Soil2 | Lime-Soil3 | Sand-Soil1 | Sand-Soil2 | Sand-Soil3 |
| --- | --- | --- | --- | --- | --- | --- | --- | --- | --- | --- | --- | --- |
| Si (Wt%) | 0.343 | 7.133 | 1.150 | 80.823 | 75.027 | 83.463 | 65.098 | 52.761 | 37.508 | 56.641 | 50.926 | 59.653 |
| Al | 0.043 | 0.330 | 0.177 | 5.823 | 10.183 | 6.547 | 4.398 | 8.093 | 2.586 | 6.542 | 4.271 | 7.024 |
| Fe | 0.110 | 0.240 | 0.133 | 3.937 | 3.900 | 3.787 | 1.738 | 3.839 | 1.124 | 3.840 | 1.737 | 3.086 |
| Na | 0.077 | 0.090 | 0.077 | 0.367 | 0.627 | 0.503 | 0.373 | 0.639 | 0.331 | 0.475 | 0.324 | 0.617 |
| Mg | 20.650 | 19.070 | 20.470 | 1.213 | 1.430 | 0.757 | 5.007 | 5.475 | 11.619 | 5.156 | 7.710 | 4.767 |
| P | 0.030 | 0.027 | 0.030 | 0.160 | 0.147 | 0.130 | 0.057 | 0.096 | 0.049 | 0.100 | 0.057 | 0.107 |
| K | 0.030 | 0.110 | 0.050 | 0.853 | 1.887 | 1.000 | 1.002 | 1.902 | 0.608 | 1.360 | 0.980 | 1.399 |
| Ca | 30.543 | 27.727 | 30.490 | 1.697 | 1.330 | 0.420 | 6.767 | 7.003 | 18.494 | 6.719 | 13.115 | 7.080 |
| Ti | 0.003 | 0.020 | 0.007 | 1.200 | 0.710 | 0.350 | 0.274 | 0.519 | 0.189 | 0.558 | 0.302 | 0.467 |
| Mn | 0.020 | 0.020 | 0.018 | 0.075 | 0.068 | 0.067 | 0.027 | 0.063 | 0.020 | 0.089 | 0.026 | 0.069 |
| V (ppm) | 6.781 | 10.424 | 7.906 | 75.543 | 49.938 | 45.807 | 29.003 | 59.503 | 27.231 | 80.374 | 36.538 | 47.950 |
| Cr | 2.339 | 7.596 | 3.332 | 65.670 | 55.736 | 34.197 | 29.082 | 63.412 | 26.874 | 84.012 | 38.022 | 50.195 |
| Co | 0.549 | 1.080 | 0.852 | 8.468 | 9.033 | 6.441 | 3.765 | 7.907 | 2.964 | 9.586 | 5.770 | 6.645 |
| Ni | 6.207 | 8.511 | 7.484 | 20.783 | 23.065 | 16.893 | 13.653 | 27.235 | 18.032 | 35.701 | 22.310 | 26.444 |
| Cu | 1.540 | 1.841 | 1.427 | 12.727 | 11.597 | 8.574 | 6.321 | 11.839 | 6.278 | 14.385 | 9.511 | 10.661 |
| Zn | 9.304 | 12.056 | 10.435 | 83.368 | 64.711 | 46.995 | 33.915 | 70.379 | 35.017 | 91.942 | 76.133 | 56.617 |
| Rb | 0.572 | 3.128 | 1.433 | 28.130 | 55.356 | 33.434 | 31.346 | 65.080 | 21.289 | 62.912 | 35.407 | 43.548 |
| Sr | 48.198 | 53.462 | 47.430 | 44.316 | 50.551 | 39.159 | 42.131 | 61.093 | 65.972 | 62.400 | 66.188 | 64.784 |
| Mo | 0.372 | 0.270 | 0.335 | 0.216 | 0.281 | 0.231 | 0.707 | 1.816 | 0.843 | 1.845 | 0.712 | 1.142 |
| Cd | 0.169 | 0.245 | 0.244 | 0.119 | 0.113 | 0.103 | 0.160 | 0.418 | 0.307 | 0.604 | 0.278 | 0.392 |
| Cs | 0.047 | 0.238 | 0.101 | 0.630 | 1.214 | 0.678 | 1.348 | 3.132 | 0.990 | 2.938 | 1.356 | 1.854 |
| Ba | 2.601 | 14.485 | 4.354 | 193.048 | 315.888 | 193.350 | 131.487 | 249.333 | 91.696 | 288.168 | 200.759 | 193.924 |
| W | 0.155 | 0.150 | 0.063 | 0.510 | 0.635 | 0.606 | 0.608 | 1.104 | 0.711 | 1.047 | 0.670 | 0.775 |

**Table S4** Pore structure characteristics of selected rock samples (n=3)

| Physical properties | Limestone1 | Limestone2 | Limestone3 | Sandstone1 | Sandstone2 | Sandstone3 | *P* values |
| --- | --- | --- | --- | --- | --- | --- | --- |
| Porosity (%) | 7.024 | 8.450 | 7.313 | 6.359 | 6.481 | 6.762 | 0.0783 |
| Mean Pore Diameter (nm) | 292.300 | 355.900 | 234.800 | 116.600 | 122.900 | 113.600 | **0.0343** |
| Median Pore Diameter (nm) | 2130.800 | 2316.300 | 2235.200 | 436.600 | 437.300 | 479.900 | **4.91E-05** |
| % pores with diameter > 1um | 23.619 | 22.341 | 19.942 | 19.693 | 20.358 | 18.681 | 0.1134 |
| % pores with diameter < 1um | 76.381 | 77.659 | 80.058 | 80.307 | 79.642 | 81.319 | 0.1134 |
| Total Water Retention Capacity [% (w/w)] | 1.015 | 1.018 | 1.014 | 1.022 | 1.022 | 1.023 | **0.0039** |

P-values shown in bold are statistically significant. P-values were calculated with the Student’s unpaired t-test.

**Table S5** Comparison of relative abundances of prokaryotic and eukaryotic taxa among different habitats

| Archaea (Phylum) | Taxonomy | Limestone (*n*=2) | Lime-Soil (*n*=5) | Sandstone (*n*=9) | Sand-Soil (*n*=7) |
| --- | --- | --- | --- | --- | --- |
|  | Euryarchaeota | 0.00 ± 0.00 b | 2.82 ± 1.54 a | 0.00 ± 0.00 b | 0.16 ± 0.11 a |
|  | Thaumarchaeota | 100.00 ± 0.00 a | 97.14 ± 1.53 b | 100.00 ± 0.00 a | 99.83 ± 0.11 ab |
|  | unclassified | 0.00 ± 0.00 | 0.02 ± 0.02 | 0.00 ± 0.00 | 0.00 ± 0.00 |
| Bacteria (Phylum) | Taxonomy | Limestone (*n*=18) | Lime-Soil (*n*=5) | Sandstone (*n*=18) | Sand-Soil (*n*=4) |
|  | Acidobacteria | 11.00 ± 2.04 b | 16.47 ± 1.47 a | 11.74 ± 3.11 b | 18.65 ± 0.91 a |
|  | Actinobacteria | 22.36 ± 6.06 a | 8.83 ± 3.22 c | 18.09 ± 5.50 ab | 13.88 ± 1.54 bc |
|  | Armatimonadetes (OP10) | 0.56 ± 0.35 b | 0.27 ± 0.19 b | 1.87 ± 1.28 a | 0.59 ± 0.20 b |
|  | Bacteroidetes | 11.58 ± 3.63 b | 17.35 ± 3.36 a | 15.92 ± 3.62 a | 10.37 ± 1.55 b |
|  | BRC1 | 0.14 ± 0.13 a | 0.10 ± 0.14 ab | 0.00 ± 0.00 b | 0.06 ± 0.13 ab |
|  | Chloroflexi | 6.54 ± 1.85 a | 2.35 ± 1.28 c | 3.97 ± 1.42 bc | 4.24 ± 0.69 b |
|  | Cyanobacteria | 9.81 ± 5.36 a | 4.15 ± 2.01 b | 10.52 ± 3.00 a | 2.80 ± 3.33 b |
|  | Deinococcus-Thermus | 0.54 ± 0.73 b | 0.01 ± 0.03 c | 0.99 ± 0.49 a | 0.00 ± 0.00 bc |
|  | Dependentiae (TM6) | 0.01 ± 0.04 a | 0.02 ± 0.05 a | 0.00 ± 0.01 a | 0.05 ± 0.10 a |
|  | Elusimicrobia | 0.01 ± 0.03 b | 0.35 ± 0.42 a | 0.00 ± 0.01 b | 0.29 ± 0.20 a |
|  | Entotheonellaeota | 0.04 ± 0.10 b | 0.96 ± 1.62 a | 0.03 ± 0.08 b | 0.66 ± 0.11 a |
|  | FBP | 2.07 ± 2.36 b | 0.07 ± 0.10 c | 3.57 ± 1.56 a | 0.22 ± 0.23 bc |
|  | FCPU426 | 0.00 ± 0.00 b | 0.02 ± 0.05 a | 0.00 ± 0.00 b | 0.00 ± 0.00 ab |
|  | Firmicutes | 0.58 ± 0.62 a | 0.58 ± 0.50 a | 0.24 ± 0.93 b | 0.33 ± 0.14 ab |
|  | Gemmatimonadetes | 1.32 ± 0.64 c | 3.01 ± 1.59 b | 1.24 ± 1.02 c | 4.39 ± 0.68 a |
|  | Hydrogenedentes | 0.00 ± 0.00 b | 0.00 ± 0.00 b | 0.00 ± 0.00 b | 0.07 ± 0.10 a |
|  | Latescibacteria | 0.01 ± 0.04 b | 5.14 ± 6.19 a | 0.00 ± 0.01 b | 2.48 ± 0.13 a |
|  | Nitrospirae | 0.03 ± 0.07 a | 0.89 ± 0.83 ab | 0.03 ± 0.05 a | 1.30 ± 0.51 b |
|  | Omnitrophicaeota | 0.00 ± 0.01 a | 0.03 ± 0.07 a | 0.00 ± 0.00 a | 0.15 ± 0.12 b |
|  | Patescibacteria | 0.23 ± 0.23 b | 0.00 ± 0.00 a | 0.38 ± 0.64 ab | 0.05 ± 0.10 ab |
|  | Planctomycetes | 7.71 ± 2.51 ab | 9.05 ± 4.54 a | 5.85 ± 2.08 b | 7.42 ± 1.23 ab |
|  | Proteobacteria | 23.61 ± 4.10 a | 27.41 ± 4.12 a | 24.02 ± 7.01 a | 29.3 ± 0.95 a |
|  | Rokubacteria | 0.00 ± 0.00 b | 0.75 ± 0.87 a | 0.01 ± 0.02 b | 1.73 ± 0.83 a |
|  | Spirochaetes | 0.00 ± 0.00 b | 0.07 ± 0.12 a | 0.00 ± 0.00 b | 0.00 ± 0.00 b |
|  | Verrucomicrobia | 1.60 ± 0.96 a | 1.71 ± 0.95 a | 1.28 ± 0.79 a | 0.77 ± 0.53 a |
|  | WPS-2 | 0.02 ± 0.03 a | 0.00 ± 0.00 a | 0.02 ± 0.04 a | 0.03 ± 0.05 a |
|  | WS2 | 0.00 ± 0.01 a | 0.00 ± 0.00 a | 0.00 ± 0.00 a | 0.03 ± 0.03 b |
|  | Zixibacteria | 0.00 ± 0.00 a | 0.00 ± 0.00 a | 0.00 ± 0.00 a | 0.00 ± 0.00 a |
|  | unclassified | 0.23 ± 0.17 | 0.40 ± 0.39 | 0.21 ± 0.24 | 0.14 ± 0.07 |
| Fungi (class) | Taxonomy | Limestone (*n*=19) | Lime-Soil (*n*=11) | Sandstone (*n*=19) | Sand-Soil (*n*=14) |
|  | Agaricomycetes | 3.81 ± 6.85 a | 8.66 ± 11.67 a | 0.06 ± 0.06 b | 3.67 ± 3.41 a |
|  | Agaricostilbomycetes | 0.00 ± 0.01 a | 0.00 ± 0.00 a | 0.00 ± 0.00 a | 0.00 ± 0.00 a |
|  | Chytridiomycetes | 0.00 ± 0.00 b | 0.01 ± 0.03 a | 0.00 ± 0.00 b | 0.00 ± 0.00 b |
|  | Cystobasidiomycetes | 0.00 ± 0.00 b | 0.24 ± 0.38 a | 0.00 ± 0.00 b | 0.03 ± 0.09 b |
|  | Dothideomycetes | 0.74 ± 0.94 b | 2.23 ± 1.81 a | 0.86 ± 0.73 ab | 2.64 ± 2.87 a |
|  | Eurotiomycetes | 18.87 ± 9.30 b | 6.68 ± 8.33 a | 12.4 ± 12.89 a | 6.44 ± 6.09 a |
|  | Geoglossomycetes | 0.00 ± 0.00 a | 0.00 ± 0.00 a | 0.00 ± 0.00 a | 0.05 ± 0.10 b |
|  | GS37 | 0.00 ± 0.00 a | 0.00 ± 0.01 a | 0.00 ± 0.00 a | 0.11 ± 0.23 b |
|  | Lecanoromycetes | 37.36 ± 15.85 b | 13.62 ± 22.44 a | 57.03 ± 21.58 b | 16.86 ± 20.61 a |
|  | Leotiomycetes | 4.74 ± 9.16 b | 33.76 ± 19.91 a | 0.83 ± 0.92 b | 28.56 ± 15.35 a |
|  | Lichinomycetes | 0.00 ± 0.00 a | 0.00 ± 0.00 ab | 0.02 ± 0.05 b | 0.00 ± 0.00 ab |
|  | Malasseziomycetes | 0.07 ± 0.11 b | 0.01 ± 0.04 a | 0.00 ± 0.00 a | 0.00 ± 0.00 a |
|  | Microbotryomycetes | 0.15 ± 0.24 a | 0.31 ± 0.43 a | 0.00 ± 0.00 b | 0.23 ± 0.42 a |
|  | Mortierellomycetes | 1.13 ± 2.24 b | 3.43 ± 3.47 a | 0.03 ± 0.03 c | 6.11 ± 3.58 a |
|  | Mucoromycetes | 0.00 ± 0.00 a | 0.00 ± 0.00 a | 0.00 ± 0.00 a | 0.00 ± 0.00 a |
|  | Orbiliomycetes | 0.00 ± 0.00 b | 0.25 ± 0.44 a | 0.00 ± 0.00 b | 0.14 ± 0.17 a |
|  | Pezizomycetes | 0.01 ± 0.02 b | 0.24 ± 0.49 a | 0.00 ± 0.00 b | 16.00 ± 26.92 a |
|  | Rhizophydiomycetes | 0.00 ± 0.00 b | 0.02 ± 0.04 a | 0.00 ± 0.00 b | 0.00 ± 0.01 ab |
|  | Rozellomycotina_cls_Incertae_sedis | 0.00 ± 0.00 a | 0.00 ± 0.00 a | 0.00 ± 0.00 a | 0.00 ± 0.00 a |
|  | Saccharomycetes | 0.03 ± 0.07 b | 0.00 ± 0.01 a | 0.00 ± 0.00 a | 0.00 ± 0.00 a |
|  | Sordariomycetes | 0.81 ± 1.11 a | 0.52 ± 0.29 ab | 0.18 ± 0.35 c | 1.89 ± 1.55 b |
|  | Tremellomycetes | 0.18 ± 0.32 ab | 0.39 ± 0.45 a | 1.85 ± 7.97 b | 0.05 ± 0.08 b |
|  | Zoopagomycetes | 0.04 ± 0.15 a | 0.00 ± 0.01 ab | 0.00 ± 0.00 b | 0.00 ± 0.00 b |
|  | unclassified | 32.05 ± 14.36 | 29.60 ± 17.01 | 26.72 ± 16.11 | 17.21 ± 9.98 |
| Protists (class) | Taxonomy | Limestone (*n*=3) | Lime-Soil (*n*=3) | Sandstone (*n*=6) | Sand-Soil (*n*=1) |
|  | Chlorodendrophyceae | 0 ± 0 | 0.33 ± 0.47 | 0 ± 0 | 0 |
|  | Chlorophyceae | 0.24 ± 0.25 | 12.26 ± 10.75 | 0 ± 0 | 0 |
|  | Chrysophyceae | 0 ± 0 | 0.21 ± 0.29 | 0 ± 0 | 0 |
|  | Granofilosea | 0 ± 0 | 0.83 ± 0.81 | 0 ± 0 | 0 |
|  | Gregarinomorphea | 1.85 ± 2.55 | 0 ± 0 | 0.35 ± 0.79 | 4.05 |
|  | Heterolobosea | 0 ± 0 | 0.03 ± 0.05 | 0 ± 0 | 4.87 |
|  | Heterotrichea | 0 ± 0 | 0.61 ± 0.45 | 0 ± 0 | 0 |
|  | Imbricatea | 0 ± 0 | 0.2 ± 0.15 | 0 ± 0 | 0 |
|  | Litostomatea | 0 ± 0 | 0 ± 0 | 0 ± 0 | 2.64 |
|  | Mycetozoa-Myxogastrea | 0 ± 0 | 0 ± 0 | 1.06 ± 0.68 | 0 |
|  | Nassophorea | 0 ± 0 | 1.13 ± 1.6 | 0 ± 0 | 0 |
|  | Oligohymenophorea | 0 ± 0 | 0 ± 0 | 0.19 ± 0.42 | 0 |
|  | Phyllopharyngea | 0.29 ± 0.43 | 0.23 ± 0.21 | 0.15 ± 0.33 | 0.83 |
|  | Proteomyxidea | 0 ± 0 | 0.96 ± 0.59 | 0 ± 0 | 1.4 |
|  | Sarcomonadea | 2.15 ± 2.2 | 25.45 ± 7.98 | 0.95 ± 0.39 | 38.89 |
|  | Spirotrichea | 0.05 ± 0.09 | 16.17 ± 1.71 | 0.57 ± 0.58 | 9.08 |
|  | Thecofilosea | 0 ± 0 | 6.7 ± 3.43 | 0.53 ± 1.09 | 1.9 |
|  | Trebouxiophyceae | 54.96 ± 23.39 | 13.37 ± 5.23 | 84.89 ± 9.06 | 20.48 |
|  | Tubulinea | 0 ± 0 | 0.32 ± 0.45 | 0 ± 0 | 0 |
|  | Ulvophyceae | 40.37 ± 25.81 | 3.56 ± 1.14 | 10.64 ± 7.75 | 0 |
|  | Variosea | 0 ± 0 | 2.1 ± 2.2 | 0 ± 0 | 0 |
|  | Xanthophyceae | 0 ± 0 | 8.9 ± 4.67 | 0 ± 0 | 0 |
|  | Unclassified | 0.09 ± 0.16 | 6.64 ± 3.98 | 0.68 ± 1.36 | 15.85 |

Values are the average (± standard deviation). Different letters in a row indicate differences between habitats (Duncan and Dunn's multiple comparison test)

**Table S6** Analysis of similarities (ANOSIM) and Permutational multivariate analysis of variance (PERMANOVA) exploring the differences in microbial community among four niches

|  | | ADONIS test | | | | | |  | ANOSIM Global test | |
| --- | --- | --- | --- | --- | --- | --- | --- | --- | --- | --- |
|  |  | Df | SumsOfSqs | MeanSqs | F.Model | *R*^2^ | *P* |  | *R* | *P* |
| Archaea | Substrate^a^ | 1 | 2.0473 | 2.0473 | 28.4510 | 0.5183 | 0.0001 |  | 0.9920 | 0.0001 |
|  | Rock type^b^ | 1 | 0.2659 | 0.2658 | 3.6946 | 0.0673 | 0.0179 |  | 0.0520 | 0.1810 |
|  | Substrate x Rock type | 1 | 0.2690 | 0.2689 | 3.7381 | 0.0681 | 0.0160 |  |  |  |
|  | Residuals | 19 | 1.3672 | 0.0719 | 0.3461 |  |  |  |  |  |
|  | Total | 22 | 3.9494 | 1.0000 |  |  |  |  |  |  |
| Bacteria | Substrate | 1 | 2.6494 | 2.6494 | 9.7917 | 0.16488 | 0.0001 |  | 0.9664 | 0.0001 |
|  | Rock type | 1 | 1.4782 | 1.4781 | 5.4631 | 0.09199 | 0.0001 |  | 0.2276 | 0.0001 |
|  | Substrate x Rock type | 1 | 0.8474 | 0.8474 | 3.1319 | 0.05274 | 0.0005 |  |  |  |
|  | Residuals | 41 | 11.0936 | 0.2705 | 0.6903 |  |  |  |  |  |
|  | Total | 44 | 16.0686 | 1.0000 |  |  |  |  |  |  |
| Fungi | Substrate | 1 | 4.2356 | 4.2356 | 15.2363 | 0.1700 | 0.0001 |  | 0.7908 | 0.0001 |
|  | Rock type | 1 | 2.6937 | 2.6937 | 9.6898 | 0.1081 | 0.0001 |  | 0.4285 | 0.0001 |
|  | Substrate x Rock type | 1 | 1.5718 | 1.5718 | 5.6540 | 0.0631 | 0.0001 |  |  |  |
|  | Residuals | 59 | 16.4018 | 0.2780 | 0.6586 |  |  |  |  |  |
|  | Total | 62 | 24.9029 | 1.0000 |  |  |  |  |  |  |
| Protists | Substrate | 1 | 1.3705 | 1.3704 | 8.1675 | 0.3589 | 0.0001 |  | 0.9999 | 0.0017 |
|  | Rock type | 1 | 0.4885 | 0.4884 | 2.9111 | 0.1279 | 0.0069 |  | 0.2328 | 0.0350 |
|  | Substrate x Rock type | 1 | 0.4490 | 0.4490 | 2.6758 | 0.1176 | 0.0147 |  |  |  |
|  | Residuals | 9 | 1.5102 | 0.1678 | 0.3955 |  |  |  |  |  |
|  | Total | 12 | 3.8181 | 1.0000 |  |  |  |  |  |  |

^a^Rock and soil as factors affecting variations in the composition of overall lithic and edaphic communities

^b^Limestone and sandstone as factors affecting variations in the composition of overall lithic and edaphic communities

**Table S7** SIMPER analysis of microbial community dissimilarity among niches. Average abundances, average dissimilarity, ratio (dissimilarity/standard deviation, Diss./SD), and percentage of cumulative similarity are also included.

|  | ASVs | Taxonomy | Closest sequence from NCBI nucleotide DB/Accession number | Similarity (%) | Av.abund. | | Av.Diss. (%) | Diss./SD | Contribution to  dissimilarity (%) | Cumulative dissimilarity (%) |
| --- | --- | --- | --- | --- | --- | --- | --- | --- | --- | --- |
|  |  |  |  |  |  |  |  |  |  |  |
|  |  |  |  |  | Lithic niches | Soil niches |  |  |  |  |
| Archaea | ASV062 | p_Thaumarchaeota; c_Nitrososphaeria; o_Nitrososphaerales; f_Nitrososphaeraceae | Uncultured archaeon clone SEV1BB071  / KJ467741 | 100 | 4.73 | 0.12 | 7.5 | 3.14 | 10.41 | 10.41 |
|  | ASV050 | p_Thaumarchaeota; c_Nitrososphaeria; o_Nitrososphaerales;  f_Nitrososphaeraceae | Unidentified archaeon FMSA14  / U62812 | 99.7 | 0 | 3.23 | 5.05 | 1.36 | 7.02 | 17.43 |
|  | ASV031 | p_Thaumarchaeota; c_Nitrososphaeria; o_Nitrososphaerales;  f_Nitrososphaeraceae | Uncultured archaeon clone SwampArchaealClone53  / MT798196 | 100 | 1.75 | 4.67 | 4.88 | 1.78 | 6.77 | 24.21 |
|  |  |  |  |  | Limestone | Sandstone |  |  |  |  |
|  | ASV036 | p_Thaumarchaeota; c_Nitrososphaeria; o_Nitrososphaerales;  f_Nitrososphaeraceae; | Uncultured archaeon clone SEV1BH011 / KJ467668 | 99.5 | 0 | 3.45 | 5.96 | 1.06 | 11.43 | 11.43 |
|  | ASV075 | p_Thaumarchaeota; c_Nitrososphaeria; o_Nitrososphaerales;  f_Nitrososphaeraceae; | Unidentified archaeon SCA1175 / U62819 | 99.7 | 2.50 | 0.45 | 4.4 | 1.18 | 8.44 | 19.87 |
|  | ASV062 | p_Thaumarchaeota; c_Nitrososphaeria; o_Nitrososphaerales;  f_Nitrososphaeraceae | Uncultured archaeon clone SEV1BB071 / KJ467741 | 100 | 3.13 | 5.08 | 3.83 | 2.00 | 7.34 | 27.21 |
|  |  |  |  |  | Lime-Soil | Sand-Soil |  |  |  |  |
|  | ASV050 | p_Thaumarchaeota; c_Nitrososphaeria; o_Nitrososphaerales;  f_Nitrososphaeraceae | Unidentified archaeon FMSA14  / U62812 | 99.7 | 4.6 | 2.24 | 4.66 | 1.55 | 10.71 | 10.71 |
|  | ASV012 | p_Thaumarchaeota; c_Nitrososphaeria; o_Nitrososphaerales;  f_Nitrososphaeraceae | Unidentified archaeon SCA1170  / U62817 | 99.7 | 1.03 | 2.31 | 3.07 | 1.87 | 7.04 | 17.75 |
|  | ASV016 | p_Thaumarchaeota; c_Nitrososphaeria; o_Nitrososphaerales;  f_Nitrososphaeraceae | Uncultured archaeon clone 355  / EF188620 | 100 | 4.25 | 2.16 | 2.98 | 2.33 | 6.84 | 24.59 |
|  |  |  |  |  | Lithic niches | Soil niches |  |  |  |  |
| Bacteria | ASV2946 | p_Gemmatimonadetes; c_Gemmatimonadetes; o_Gemmatimonadales;  f_Gemmatimonadaceae | Uncultured bacterium clone 5A_10-065  / KY190543 | 99.5 | 0.13 | 2.02 | 1.53 | 0.83 | 1.56 | 1.56 |
|  | ASV8674 | p_Gemmatimonadetes; c_Gemmatimonadetes; o_Gemmatimonadales;  f_Gemmatimonadaceae | Uncultured bacterium clone 5A_10-059  / KY190537 | 99.5 | 0 | 1.89 | 1.37 | 1.26 | 1.4 | 2.96 |
|  | ASV3877 | p_Gemmatimonadetes; c_Gemmatimonadetes; o_Gemmatimonadales;  f_Gemmatimonadaceae | Uncultured bacterium clone 5A_10-024  / KY190513 | 99.5 | 0 | 1.68 | 1.21 | 1.25 | 1.24 | 4.2 |
|  |  |  |  |  | Limestone | Sandstone |  |  |  |  |
|  | ASV9058 | p_Chloroflexi; c_Chloroflexia; o_Thermomicrobiales;  f_JG30-KF-CM45 | Uncultured bacterium clone Ovdat-33  / GQ425264 | 99.5 | 0.85 | 0.35 | 0.38 | 1.18 | 0.46 | 0.46 |
|  | ASV0793 | p_Actinobacteria; c_Thermoleophilia; o_Solirubrobacterales; f_Solirubrobacteraceae | Uncultured bacterium clone UMAB-cl-17  / FN811201 | 99.5 | 0.66 | 0.64 | 0.37 | 0.69 | 0.45 | 0.91 |
|  | ASV7996 | p_Actinobacteria; c_Thermoleophilia; o_Solirubrobacterales; f_67-14 | Uncultured Rubrobacteridae bacterium  / HE974791 | 98.0 | 0.73 | 0.18 | 0.33 | 1.2 | 0.4 | 1.31 |
|  |  |  |  |  | Lime-Soil | Sand-Soil |  |  |  |  |
|  | ASV2400 | p_Proteobacteria; c_Gammaproteobacteria; o_Betaproteobacteriales;  f_Burkholderiaceae; g_Rhizobacter | Burkholderiales bacterium Se3Ps_2574  / AB835073 | 99.7 | 2.00 | 0 | 4.28 | 0.49 | 5.18 | 5.18 |
|  | ASV6620 | p_Acidobacteria; c_Subgroup 6 | Uncultured bacterium clone 7A_10-030  / KY190684 | 99.2 | 0 | 2.56 | 4.11 | 1.35 | 4.98 | 10.16 |
|  | ASV4279 | p_Entotheonellaeota; c_Entotheonellia; o_Entotheonellales;  f_Entotheonellaceae | Uncultured bacterium clone 3.23  / KR560030 | 99.5 | 0 | 2.58 | 4.04 | 1.49 | 4.89 | 15.04 |
|  |  |  |  |  | Lithic niches | Soil niches |  |  |  |  |
| Fungi | ASV3498 | p_Ascomycota; c_Leotiomycetes; o_Thelebolales;  f_Pseudeurotiaceae; g_Geomyces | Geomyces sp.  / KU556580 | 100 | 0.01 | 4.15 | 3.22 | 2.23 | 3.36 | 3.36 |
|  | ASV0226 | p_Ascomycota; c_Pezizomycetes; o_Pezizales;  f_Pyronemataceae; g_Pulvinula | Uncultured Pulvinula isolate 5-1-8-n3  / MK614840 | 97.5 | 0.01 | 1.30 | 1.02 | 0.47 | 1.07 | 4.42 |
|  | ASV2663 | p_Mortierellomycota ;c_Mortierellomycetes; o_Mortierellales;  f_Mortierellaceae; g_Mortierella | Mortierella antarctica GR3-2-4-8  / LC515021 | 100 | 0.01 | 1.25 | 0.99 | 1.22 | 1.03 | 5.45 |
|  |  |  |  |  | Limestone | Sandstone |  |  |  |  |
|  | ASV2293 | p_Ascomycota; c_Lecanoromycetes; o_Hymeneliales;  f_Hymeneliaceae; g_Hymenelia | Hymenelia melanocarpa isolate 1046  / KJ462266 | 99.2 | 2.29 | 0.05 | 1.57 | 1.34 | 1.67 | 1.67 |
|  | ASV0935 | p_Ascomycota; c_Lecanoromycetes; o_Lecanorales; | Lecidea sp. voucher J. Malicek 10656  / MK778613 | 89.4 | 0.05 | 1.95 | 1.38 | 1.52 | 1.47 | 3.14 |
|  | ASV0827 | p_Ascomycota; c_Lecanoromycetes; o_Lecanorales;  f_Lecanoraceae; g_Lecidella | Lecidella carpathica  / DQ534471 | 98.8 | 0.00 | 1.77 | 1.27 | 0.75 | 1.35 | 4.49 |
|  |  |  |  |  | Lime-Soil | Sand-Soil |  |  |  |  |
|  | ASV0226 | p_Ascomycota; c_Pezizomycetes; o_Pezizales;  f_Pyronemataceae; g_Pulvinula | Uncultured Pulvinula isolate 5-1-8-n3  / MK614840 | 97.5 | 0.00 | 2.32 | 1.94 | 0.68 | 2.40 | 2.4 |
|  | ASV3498 | p_Ascomycota; c_Leotiomycetes; o_Thelebolales;  f_Pseudeurotiaceae; g_Geomyces | Geomyces sp.  / KU556580 | 100 | 3.80 | 4.42 | 1.79 | 1.24 | 2.22 | 4.62 |
|  | ASV1756 | p_Ascomycota; c_Leotiomycetes; o_Helotiales; | Calycellina triseptata strain CBS 606.77  / MH861105 | 96.0 | 1.55 | 0.00 | 1.43 | 0.51 | 1.77 | 6.39 |
|  |  |  |  |  | Lithic niches | Soil niches |  |  |  |  |
| Protists | ASV217 | Archaeplastida | Uncultured Chytridiomycota clone T1P1AeH05  / GQ995375 | 99.7 | 3.55 | 0.14 | 3.2 | 1.92 | 3.38 | 3.38 |
|  | ASV304 | p_Cercozoa; c_Sarcomonadea; o_Cercomonadida;  f_Cercomonadidae; g_Cercomonas | Pseudophialophora sp. isolate LJJ23  / MT379655 | 96.5 | 3.25 | 0.00 | 3.06 | 1.3 | 3.23 | 6.61 |
|  | ASV112 | p_Cercozoa; c_Imbricatea; o_Plasmodiophorida | Uncultured soil eukaryote clone a411  / MK945971 | 97.5 | 0.00 | 2.51 | 2.46 | 1.94 | 2.60 | 9.22 |
|  |  |  |  |  | Limestone | Sandstone |  |  |  |  |
|  | ASV304 | p_Cercozoa; c_Sarcomonadea; o_Cercomonadida;  f_Cercomonadidae; g_Cercomonas | Pseudophialophora sp. isolate LJJ23  / MT379655 | 96.5 | 0.60 | 4.57 | 5.01 | 2.08 | 7.45 | 7.45 |
|  | ASV249 | p_Apicomplexa; c_Conoidasida; o_Eugregarinorida | Uncultured eukaryote clone OTU528  / MT531329 | 95.4 | 4.13 | 0.67 | 4.4 | 2.26 | 6.53 13.98 | 13.98 |
|  | ASV084 | p_Cercozoa; c_Sarcomonadea; o_Glissomonadida | Uncultured Cryomonadida clone Fr_Dv_411  / KT251194 | 99.7 | 2.75 | 0.50 | 3.17 | 1.53 | 4.71 18.69 | 18.69 |
|  |  |  |  |  | Lime-Soil | Sand-Soil |  |  |  |  |
|  | ASV150 | p_Cercozoa; c_Proteomyxidea; o_Aconchulinida;  f_Vampyrellida; g_Vampyrellida clade B5 | Uncultured soil eukaryote clone f319  / MK946106 | 73.1 | 0.00 | 3.44 | 2.58 | 5.92 | 2.98 | 2.98 |
|  | ASV314 | p_Chlorophyta; c_Chlorophyceae; o_Chlamydomonadales;  f_Haematococcaceae; g_Stephanosphaera | Uncultured eukaryote clone HRT3hrs_A5_euk_7  / LC222955 | 78.5 | 2.88 | 0.00 | 2.27 | 1.33 | 2.62 | 5.6 |
|  | ASV153 | p_Cercozoa; c_Thecofilosea | Uncultured eukaryote clone Elev_18S_5021  / EF025005 | 99.5 | 2.99 | 0.00 | 2.24 | 2.79 | 2.59 | 8.2 |

**Table S8** ASVs compositions of specialists, generalists and unspecified taxa within each bacterial phylum and fungal classes

| Domain | Taxonomy | Generalists | Specialists | | Unspecified |
| --- | --- | --- | --- | --- | --- |
|  |  |  | Lithic niches | Edaphic niches |  |
| Bacteria | Acidobacteria | 20 | 9 | 7 | 25 |
|  | Actinobacteria | 85 | 0 | 6 | 22 |
|  | Armatimonadetes | 0 | 2 | 0 | 0 |
|  | Bacteroidetes | 31 | 15 | 9 | 4 |
|  | Chloroflexi | 11 | 1 | 0 | 3 |
|  | Cyanobacteria | 43 | 3 | 1 | 15 |
|  | Deinococcus-Thermus | 0 | 1 | 0 | 2 |
|  | Entotheonellaeota | 0 | 0 | 1 | 1 |
|  | FBP | 13 | 2 | 0 | 1 |
|  | Gemmatimonadetes | 4 | 0 | 1 | 3 |
|  | Latescibacteria | 0 | 0 | 4 | 2 |
|  | Nitrospirae | 0 | 0 | 1 | 0 |
|  | Planctomycetes | 5 | 3 | 3 | 5 |
|  | Proteobacteria | 85 | 1 | 14 | 27 |
|  | Rokubacteria | 0 | 0 | 0 | 1 |
|  | Verrucomicrobia | 7 | 0 | 0 | 1 |
| Fungi | Agaricomycetes | 6 | 0 | 3 | 16 |
|  | Cystobasidiomycetes | 0 | 0 | 0 | 1 |
|  | Dothideomycetes | 6 | 3 | 3 | 2 |
|  | Eurotiomycetes | 31 | 15 | 3 | 30 |
|  | Lecanoromycetes | 31 | 16 | 5 | 63 |
|  | Leotiomycetes | 29 | 0 | 6 | 8 |
|  | Microbotryomycetes | 1 | 0 | 1 | 0 |
|  | Mortierellomycetes | 9 | 0 | 1 | 4 |
|  | Orbiliomycetes | 0 | 0 | 0 | 1 |
|  | Pezizomycetes | 1 | 0 | 1 | 2 |
|  | Sordariomycetes | 5 | 0 | 4 | 3 |
|  | Tremellomycetes | 0 | 1 | 0 | 1 |
|  | unclassified | 79 | 16 | 20 | 70 |

**Table S9** Topological attributes of the co-occurrence networks of bacterial and fungal communities in different rock types

|  | No. of  Nodes | No. of  Edges | Average  degree | Modularity | Clustering  coefficient | Average  path length | Network  diameter | Graph  density | positive/negative  edge ratio |
| --- | --- | --- | --- | --- | --- | --- | --- | --- | --- |
| Limestone | 146 | 197 | 2.699 | 0.709 | 0.101 | 5.582 | 14 | 0.019 | 2.17 |
| Sandstone | 157 | 255 | 3.248 | 0.613 | 0.069 | 4.841 | 11 | 0.021 | 1.52 |

**Table S10**. Numbers of links in the networks obtained from Limestone and Sandstone. The percentages in brackets represent the proportion of each number of links relative to the total links for each niche.

|  | Limestone | Sandstone |
| --- | --- | --- |
| Total links | 197 | 255 |
| Positive links | 135 (68.5%) | 154 (60.4%) |
| Negative links | 62 (31.5%) | 101 (39.6%) |
|  |  |  |
| Bacteria-Bacteria |  |  |
| Positive links | 54 (27.4%) | 69 (27.1%) |
| Negative links | 22 (11.2%) | 42 (16.5%) |
|  |  |  |
| Fungi-Fungi |  |  |
| Positive links | 54 (27.4%) | 53 (20.7%) |
| Negative links | 26 (13.2%) | 30 (11.8%) |
|  |  |  |
| Bacteria-Fungi |  |  |
| Positive links | 27 (13.7%) | 32 (12.5%) |
| Negative links | 14 (7.1%) | 29 (11.4%) |

**Table S11** Taxonomic affiliations of top five ASVs as keystone taxa defined by node degree and betweenness

| Rock type | ASVs | No. of degree | Betweenness | Kingdom | Phylum | Class | Order | Family | Genus | Species |  |
| --- | --- | --- | --- | --- | --- | --- | --- | --- | --- | --- | --- |
| Limestone | ASV3249 | 7 | 1585.5 | Fungi | Ascomycota | Unclassified | Unclassified | Unclassified | Unclassified | Unclassified |  |
|  | ASV1680 | 6 | 1944.3 | Fungi | Ascomycota | Unclassified | Unclassified | Unclassified | Unclassified | Unclassified |  |
|  | ASV2646 | 6 | 1600.4 | Fungi | Ascomycota | Eurotiomycetes | Verrucariales | Verrucariaceae | Polyblastia | Unclassified |  |
|  | ASV1742 | 6 | 1061.9 | Fungi | Ascomycota | Eurotiomycetes | Verrucariales | Verrucariaceae | Verrucaria | Unclassified |  |
|  | ASV0849 | 6 | 1056.7 | Bacteria | Actinobacteria | Thermoleophilia | Solirubrobacterales | Conexibacteraceae | Conexibacter | Unclassified |  |
| Sandstone | ASV0165 | 9 | 1136.6 | Fungi | Ascomycota | Eurotiomycetes | Verrucariales | Verrucariaceae | Unclassified | Unclassified |  |
|  | ASV2011 | 8 | 1167.2 | Fungi | Ascomycota | Lecanoromycetes | Lecanorales | Porpidiaceae | Porpidia | Unclassified |  |
|  | ASV0659 | 6 | 1188.6 | Fungi | Ascomycota | Lecanoromycetes | Candelariales | Candelariaceae | Candelariella | Candelariella_xanthostigma |  |
|  | ASV1105 | 6 | 1072.8 | Fungi | Basidiomycota | Tremellomycetes | Tremellales | Tremellaceae | Unclassified | Unclassified |  |
|  | ASV6009 | 6 | 1041.0 | Bacteria | Actinobacteria | Thermoleophilia | Solirubrobacterales | Conexibacteraceae | Conexibacter | Unclassified |  |

**2. Supplementary Methods**

This part includes a metadata table to link the SRR codes from the BioProject to the samples and the commands used to construct ASV tables for archaea, bacteria, fungi, and protists. Additionally, it includes the R scripts used for the statistical analyses.

**Metadata table**

| Accession | Study | Bioproject  accession | Biosample  accession | Library strategy | Library layout | Instrument model | Design description | File type | File name | File name2 |
| --- | --- | --- | --- | --- | --- | --- | --- | --- | --- | --- |
| SRR9914652 | SRP102559 | PRJNA380676 | SAMN12501501 | AMPLICON | paired | Illumina MiSeq | Archaea-V6-V8 | fastq | Archaea_1.fastq.gz | Archaea_2.fastq.gz |
| SRR9914651 | SRP102559 | PRJNA380676 | SAMN12501501 | AMPLICON | paired | Illumina MiSeq | Bacteria-V4-V5 | fastq | Bacteria_1.fastq.gz | Bacteria_2.fastq.gz |
| SRR9914654 | SRP102559 | PRJNA380676 | SAMN12501501 | AMPLICON | paired | Illumina MiSeq | Eukaryote-V4 | fastq | Eukaryotes_1.fastq.gz | Eukaryotes_2.fastq.gz |
| SRR9914653 | SRP102559 | PRJNA380676 | SAMN12501501 | AMPLICON | paired | Illumina MiSeq | Fungi-ITS2 | fastq | Fungi_1.fastq.gz | Fungi_2.fastq.gz |

**2-1. Inference of Amplicon Sequence Variants (ASVs)**

***#Inspect read quality***

mkdir fastqc_out

fastqc -t 20 raw_data/*.fastq.gz -o fastqc_out

cd fastqc_out

multiqc .

***#Activate QIIME2***

***#Import***

mkdir reads_qza

qiime tools import \

--type SampleData[PairedEndSequencesWithQuality] \

--input-path raw_data/ \

--output-path reads_qza/reads.qza \

--input-format CasavaOneEightSingleLanePerSampleDirFmt

***#Trim primers with cutadapt***

qiime cutadapt trim-paired \

--i-demultiplexed-sequences reads_qza/reads.qza \

--p-cores 20 \

--p-front-f * \

--p-front-r * \

--p-discard-untrimmed \

--p-no-indels \

--o-trimmed-sequences reads_qza/reads_trimmed.qza

*Archaea F: TYAATYGGANTCAACRCC / R:CRGTGWGTRCAAGGRGCA

*Bacteria F: GTGYCAGCMGCCGCGGTAA / R: CCGYCAATTYMTTTRAGTTT

*Fungi F: GTGAATCATCGAATCTTTGAA / R: TCCTCCGCTTATTGATATGC

*Eukaryotes F: CYGCGGTAATTCCAGCTC / R: AYGGTATCTRATCRTCTTYG

***#Denoising***

qiime dada2 denoise-paired --i-demultiplexed-seqs 3.reads_qza/reads_trimmed.qza --p-trunc-len-f * --p-trunc-len-r * --p-max-ee-f 3 --p-max-ee-r 7 --p-n-threads 20 --output-dir 4.dada2_output

*Archaea F: 240 / R: 200

*Bacteria F: 270 / R: 230

*Fungi F: 250 / R: 220

*Eukaryotes F: 260 / R: 220

qiime tools export --input-path 4.dada2_output/denoising_stats.qza --output-path 4.dada2_output

qiime feature-table summarize --i-table 4.dada2_output/table.qza --o-visualization 4.dada2_output/dada2_table_summary.qzv

***#taxonomic classification***

qiime feature-classifier classify-sklearn --i-reads 4.dada2_output/representative_sequences.qza --i-classifier *_classifier.qza --p-n-jobs 20 --output-dir 5.taxa

*Archaea: silva_132_99_16S_V6.V8_A956F_A1401R.qza

*Bacteria: silva_132_99_16S_V4.V5_515F_926R.qza

*Fungi: sh_refs_qiime_ver8_99_s_all_02.02.2019_ITS.qza

*Protists: pr2_4.12.0_18S_classifier.qza

qiime tools export --input-path 5.taxa/classification.qza --output-path 5.taxa

***#filter out rare ASVs***

qiime feature-table filter-features --i-table 4.dada2_output/table.qza --p-min-frequency * --p-min-samples 1 --o-filtered-table 4.dada2_output/dada2_table_filt.qza

*Archaea: 17 / Bacteria: 20 / Fungi: 30 / Protists: 12

***#filter out contaminant and unclassified ASVs***

qiime taxa filter-table --i-table 4.dada2_output/dada2_table_filt.qza --i-taxonomy 5.taxa/classification.qza --p-exclude * --o-filtered-table 4.dada2_output/dada2_table_filt_contam.qza

*Archaea: mitochondria,chloroplast,Bacteria,Unassigned

*Bacteria: Archaea,mitochondria,chloroplast,Unassigned

*Fungi: Unassigned,Viridiplantae,Protista,Rhizaria

*Protists: Fungi,Metazoa,Streptophyta

***#exclude low-depth samples***

qiime feature-table filter-samples --i-table 4.dada2_output/dada2_table_filt_contam.qza --p-min-frequency * --o-filtered-table 4.dada2_output/dada2_table_final.qza

*Archaea: 4000 / Bacteria: 3000 / Fungi: 2000 / Protists: 1000

***#Generate rarefaction curves***

qiime diversity alpha-rarefaction --i-table 4.dada2_output/dada2_table_final.qza --p-max-depth * --p-steps 20 --i-phylogeny asvs-tree.qza --m-metadata-file metadata.txt --o-visualization rarefaction_curves_*.qzv

*Archaea: 4000 / Bacteria: 3000 / Fungi: 2000 / Protists: 1000

***#Generate stacked barchart of taxa relative abundances***

qiime taxa barplot --i-table 4.dada2_output/dada2_table_final.qza --i-taxonomy 5.taxa/classification.qza --m-metadata-file metadata.txt --o-visualization 5.taxa/taxa_barplot.qzv

***#Calculating diversity metrics and generating ordination plots***

qiime diversity core-metrics-phylogenetic --i-table 4.dada2_output/dada2_table_final.qza --i-phylogeny asvs-tree.qza --p-sampling-depth * --m-metadata-file metadata.txt --p-n-jobs-or-threads 20 --output-dir 6.diversity_*

*Archaea: 4000 / Bacteria: 3000 / Fungi: 2000 / Protists: 1000

#Exporting the final profile and sequence files

qiime tools export --input-path 4.dada2_output/rep_seqs_final.qza --output-path 7.dada2_output_exported

qiime tools export --input-path 4.dada2_output/dada2_table_final.qza --output-path 7.dada2_output_exported

**2-2. R scripts used for the statistical analyses (RStudio version R 3.6.1)**

***#NMDS***

getwd()

setwd("D:/~/nmds ")

library(vegan)

library(ggplot2)

library(extrafont)

library(metagenomeSeq)

arc = read.csv("arc.csv",header = T) #bacteria, fungi, and protists were analyzed in the same way

a.com = arc[,4:ncol(arc)]

a.m_com = as.matrix(a.com)

t.a.m_com <- decostand(a.m_com, method = "hellinger")

a.nmds = metaMDS(t.a.m_com, distance = "bray")

a.nmds

goodness(a.nmds)

stressplot(a.nmds)

a.data.scores = as.data.frame(scores(a.nmds))

a.data.scores$Samples = arc$Samples

a.data.scores$group = arc$group

a.data.scores$type = arc$type

head(a.data.scores)

a.xx = ggplot(a.data.scores, aes(x = NMDS1, y = NMDS2)) +

geom_hline(yintercept=0, linetype='dashed', color='grey', size=0.5)+

geom_vline(xintercept=0, linetype='dashed', color='grey', size=0.5)+

geom_point(size = 6, aes(shape = type, fill=type), colour = "black")+

theme(axis.text.y = element_text(colour = "black", size = 23, family="Times New Roman"),

axis.text.x = element_text(colour = "black", size = 23, family="Times New Roman"),

legend.text = element_text(size = 12, colour ="black", family="Times New Roman"),

legend.position = "right", axis.title.y = element_text(size = 25),

axis.title.x = element_text(size = 25, colour = "black", family="Times New Roman"),

legend.title = element_text(size = 20, colour = "black", family="Times New Roman"),

panel.background = element_blank(), panel.border = element_rect(colour = "black", fill = NA),

legend.key=element_blank()) +

labs(x = "NMDS1", colour = "type", y = "NMDS2", shape = "type")+

scale_shape_manual(values = c(22,21,22,21))+

scale_fill_manual(values = c("#FF7F50", "#FF0000", "#00BFFF", "#0000FF"))

a.xx

***#PERMDISP***

library(biomformat)

library(GGally)

library(dplyr)

library(tidyr)

library(grid)

library(gridExtra)

library(ggplot2)

library(ggvegan)

library(vegan)

source("C:/Users/phyloramalina_post/Desktop/antarctic_glacier/R/cleanplot.pca.R")

source("C:/Users/KOPRI/Desktop/nmds/myplotbetadisp.R")

library(githubinstall)

library(ggfortify)

library(betapart)

library(picante)

library(ape) source("myplotbetadisp.r")

arc = read.csv("arc.csv",header = T) #bacteria, fungi, and protists were analyzed in the same way

a.m_com = as.data.frame(arc)

a.m_com <- as.matrix(sapply(a.m_com, as.numeric))

a.m_com <- newMRexperiment(a.m_com)

t.a.m_com = cumNorm(a.m_com, p=cumNormStatFast(a.m_com))

arc_CSS = data.frame(MRcounts(t.a.m_com, norm=TRUE, log=TRUE))

arc_meta = read.csv("arc_metadata.csv",header = T)

str(arc_meta)

#betadisper#

a.groups_rs <- factor(c(rep(1,11), rep(2,12)), labels = c("Rock","Soil"))

a.dis_rs <- vegdist(arc_CSS, "bray")

a.mod_rs <- betadisper(a.dis_rs, a.groups_rs)

a.mod_rs

anova(a.mod_rs)

permutest(a.mod_rs, pairwise = TRUE, permutations = 99)

(a.mod_rs.HSD <- TukeyHSD(a.mod_rs))

myplotbetadisper(a.mod_rs, ellipse = FALSE, hull = TRUE,main= "a.MultiVariate Permutation RS")

boxplot(a.mod_rs)

***#ANOSIM/ADONIS***

getwd()

setwd("D:/Research/2016_rock_data/2016_amplicon data/adonis")

path<-"D:/Research/2016_rock_data/2016_amplicon data/adonis"

library("vegan")

arc <- read.csv("D:/Research/2016_rock_data/2016_amplicon data/adonis/arc_input.csv", header = TRUE) #bacteria, fungi, and protists were analyzed in the same way

arc

a_substrate=as.factor(arc[,2])

a_type=as.factor(arc[,3])

a_otu=as.matrix(arc[,4:85])

adonis(formula = a_otu~a_substrate*a_type, distance = "bray", permutation = 9999)

adonis(formula = a_otu~a_substrate, distance = "bray", permutation = 9999)

adonis(formula = a_otu~a_type, distance = "bray", permutation = 9999)

anosim(arc[,4:85], grouping = arc$substrate, distance = "bray", permutation = 9999)

anosim(arc[,4:85], grouping = arc$type, distance = "bray", permutation = 9999)

***#Network analysis***

library(ggplot2)

library(igraph)

library(Matrix)

library(SpiecEasi)

library(phyloseq)

library(ggpubr)

library(tidyverse)

library(Hmisc)

library(corrplot)

library(microbiome)

library(devtools)

getwd()

setwd("~/Spieceasi_multicross")

Packages <- c("phyloseq", "data.table", "ggplot2", "plyr","dplyr","reshape2","grid",

"gridExtra","scales","dplyr", "ggpubr","vegan","multcompView","rcompanion","betapart")

lapply(Packages, library, character.only = TRUE)

asv_mat<- read.csv("Bact_OTU.csv")

tax_mat<- read.csv("Bact_TAX.csv")

samples_df <- read.csv("Bact_samdt.csv")

row.names(asv_mat) <- asv_mat$ASV

asv_mat <- asv_mat %>% select (-ASV)

row.names(tax_mat) <- tax_mat$ASV

tax_mat <- tax_mat %>% select (-ASV)

row.names(samples_df) <- samples_df$sample

samples_df <- samples_df %>% select (-sample)

asv_mat <- as.matrix(asv_mat)

tax_mat <- as.matrix(tax_mat)

OTU = otu_table(asv_mat, taxa_are_rows = TRUE)

TAX = tax_table(tax_mat)

samples = sample_data(samples_df)

Bact <- phyloseq(OTU, TAX, samples)

Bact

f.asv_mat<- read.csv("Fung_OTU_same_bac_samples.csv")

f.tax_mat<- read.csv("Fung_TAX.csv")

f.samples_df <- read.csv("Fung_samdt_same_bac_samples.csv")

row.names(f.asv_mat) <- f.asv_mat$ASV

f.asv_mat <- f.asv_mat %>% select (-ASV)

row.names(f.tax_mat) <- f.tax_mat$ASV

f.tax_mat <- f.tax_mat %>% select (-ASV)

row.names(f.samples_df) <- f.samples_df$sample

f.samples_df <- f.samples_df %>% select (-sample)

f.asv_mat <- as.matrix(f.asv_mat)

f.tax_mat <- as.matrix(f.tax_mat)

f.OTU = otu_table(f.asv_mat, taxa_are_rows = TRUE)

f.TAX = tax_table(f.tax_mat)

f.samples = sample_data(f.samples_df)

Fung <- phyloseq(f.OTU, f.TAX, f.samples)

Fung

#filter by relative abundance

Bactfilt <- transform_sample_counts(Bact,function(x) ifelse(x>=0.001*sum(x),x,0))

condition <- function(x) { x > 0 }

taxaToKeep <- genefilter_sample(Bactfilt, condition, 1)

Bactall<-prune_taxa(taxaToKeep, Bactfilt)

Fungfilt <- transform_sample_counts(Fung,function(x) ifelse(x>=0.001*sum(x),x,0))

condition <- function(x) { x > 0 }

taxaToKeep <- genefilter_sample(Fungfilt, condition, 1)

Fungall<-prune_taxa(taxaToKeep, Fungfilt)

x<-data.frame()

COND<-list('Limestone','Sandstone')

for (i in COND)

Bact<-subset_samples(Bactall, group == i)

Fung<-subset_samples(Fungall, group == i)

condition <- function(x) { x > 0 }

taxafilt <- genefilter_sample(Bact, condition, 1)

Bact<-prune_taxa(taxafilt, Bact)

taxafilt <- genefilter_sample(Fung, condition, 1)

Fung<-prune_taxa(taxafilt, Fung)

taxafilt <- genefilter_sample(Bact, condition, 6)

Bact.occ8<-prune_taxa(taxafilt, Bact)

taxafilt <- genefilter_sample(Fung, condition, 6)

Fung.occ8<-prune_taxa(taxafilt, Fung)

sample_names(Fung.occ8)

sample_names(Bact.occ8)

x<-data.frame()

COND<-list('Sandstone','Limestone')

for (i in COND)

Bact<-subset_samples(Bactall, group == i)

Fung<-subset_samples(Fungall, group == i)

condition <- function(x) { x > 0 }

taxafilt <- genefilter_sample(Bact, condition, 1)

Bact<-prune_taxa(taxafilt, Bact)

taxafilt <- genefilter_sample(Fung, condition, 1)

Fung<-prune_taxa(taxafilt, Fung)

taxafilt <- genefilter_sample(Bact, condition, 6)

Bact.occ8<-prune_taxa(taxafilt, Bact)

taxafilt <- genefilter_sample(Fung, condition, 6)

Fung.occ8<-prune_taxa(taxafilt, Fung)

sample_names(Fung.occ8)

sample_names(Bact.occ8)

# network#

spiec <- spiec.easi(list(Bact.occ8, Fung.occ8), method='mb', nlambda=100,

lambda.min.ratio=1e-2, pulsar.params = list(thresh = 0.05))

dtypex <- c(rep(1,ntaxa(Bact.occ8)), rep(2,ntaxa(Fung.occ8)))

dtype <- c(taxa_names(Bact.occ8), taxa_names(Fung.occ8))

list(name=dtype)

ig<-adj2igraph(symBeta(getOptBeta(spiec)))

igRefit<-adj2igraph(getRefit(spiec))

CoordDelete <- layout_nicely(igRefit)

nodedegree<-(degree(ig)==0)

names(nodedegree)<-dtype

NodeInteracting<-table(nodedegree)["FALSE"]

NodeNonInteracting<-table(nodedegree)["TRUE"]

#network with weight#

#pdf(paste0(path2,i,".pdf"), width = 30,height = 30)

set.seed(1)

plot(delete.vertices((adj2igraph(getRefit(spiec), vertex.attr=list(name=dtype,color=dtypex,size=10,label.cex=0.8),

edge.attr=list(width=5,color=(ifelse(E(ig)$weight > 0.0000, 'green', 'red'))))),names(which(nodedegree == "TRUE"))),)+title(i,cex.main = 3)

spiec.graph=adj2igraph(getRefit(spiec), vertex.attr=list(name=dtype))

output.path="D:/Research/2016_rock_data/2016_amplicon data/network_analysis/network_2019~/Spieceasi_multicross"

write.graph(spiec.graph,file=file.path(output.path,"lime_qqqqq_ncol.txt"),format="ncol")
